# The 17D-204 Vaccine Strain-Induced Protection against Virulent Yellow Fever Virus Is Mediated by Humoral Immunity and CD4+ but not CD8+ T Cells

**DOI:** 10.1371/journal.ppat.1005786

**Published:** 2016-07-27

**Authors:** Alan M. Watson, L. K. Metthew Lam, William B. Klimstra, Kate D. Ryman

**Affiliations:** Center for Vaccine Research, Department of Microbiology and Molecular Genetics, University of Pittsburgh, Pittsburgh, Pennsylvania, United States of America; NIH, UNITED STATES

## Abstract

A gold standard of antiviral vaccination has been the safe and effective live-attenuated 17D-based yellow fever virus (YFV) vaccines. Among more than 500 million vaccinees, only a handful of cases have been reported in which vaccinees developed a virulent wild type YFV infection. This efficacy is presumed to be the result of both neutralizing antibodies and a robust T cell response. However, the particular immune components required for protection against YFV have never been evaluated. An understanding of the immune mechanisms that underlie 17D-based vaccine efficacy is critical to the development of next-generation vaccines against flaviviruses and other pathogens. Here we have addressed this question for the first time using a murine model of disease. Similar to humans, vaccination elicited long-term protection against challenge, characterized by high neutralizing antibody titers and a robust T cell response that formed long-lived memory. Both CD4+ and CD8+ T cells were polyfunctional and cytolytic. Adoptive transfer of immune sera or CD4+ T cells provided partial protection against YFV, but complete protection was achieved by transfer of both immune sera and CD4+ T cells. Thus, robust CD4+ T cell activity may be a critical contributor to protective immunity elicited by highly effective live attenuated vaccines.

## Author Summary

The 17D line yellow fever virus (YFV) vaccines are some of the safest and most effective live-attenuated virus vaccines ever produced, protecting recipients for life against deadly yellow fever (YF). As a testament to this safety and efficacy, the 17D line of live-attenuated vaccines has become an important model for the design of future vaccines. However, we still lack a fundamental understanding of the protective immunity elicited against the virulent YFV, a knowledge gap that must be overcome to inform the design of future live-attenuated and subunit vaccines. Humans develop robust antibody and T cell responses following vaccination, leading some to suggest that vaccine-elicited CD8+ T cells are important for protection against virulent YFV. Since this can never be tested in humans, we have used mice to model immunity to the 17D-204 vaccine strain. We found that CD4+ T cells elicited by 17D-204 contributed to protection against virulent YFV, but CD8+ T cells had no effect on the outcomes of survival or disease. Our study is the first to demonstrate that vaccine-elicited CD4+ T cells can protect against YFV infection. These results suggest that vaccine developers should consider the importance of CD4+ T cells when designing vaccines against viruses similar to YFV.

## Introduction

Live-attenuated vaccines (LAV) generally provide the highest level of protection against infectious diseases. The most effective LAVs duplicate the pathogen-specific conditions of natural infection but have their replication curtailed by the innate and adaptive immune responses prior to the onset of clinical disease. A well-balanced combination of authentic antigen expression and control can induce a prolific adaptive immune response and the formation of long-lived memory. The development of LAVs is generally a results-driven empirical process controlling first for attenuation and subsequently for protection. Although the broad immunological response to these vaccines is often times examined exquisitely, the immunity that directly contributes to protection is more difficult to define. Exploring the protective immunity elicited by LAVs would require the use of human subjects, which is often not appropriate, or animal model systems which may not accurately represent immunity or disease. However, understanding the immune properties that are required for protection is crucial to the rational design of vaccines against pathogens for which empirical production of a LAV has failed or for which usage of a LAV is prevented by current vaccine standards.

One of the most successful lines of LAVs uses the 17D-based vaccine strains of yellow fever virus (YFV). Since its introduction in the 1930s [1] the 17D-based vaccines (substrains 17D-204 and 17DD) have proven themselves to be amongst the most successful and efficacious vaccines created [2]. Centuries prior to the introduction of the 17D line of YFV vaccines, yellow fever (YF) was one of the most feared and widespread epidemic diseases in Africa, Europe, and the Americas. More than 500 million people have been vaccinated with the 17D-based vaccines and only 12 known cases of vaccine failure have resulted in YF [3]. The 17D line of vaccines also has an excellent safety record resulting in extremely rare severe adverse events [4]. Immunization with the 17D line of vaccines remains a mainstay in the YF endemic zones of Central/South America and Sub-Saharan Africa where sylvatic YFV reservoirs still seed endemic disease and outbreaks, offering the only protection to over 900 million people world-wide [5]. The strong and enduring response from a single vaccination has led to the recommendation that only a single dose is required for life-long immunity [6,7]. As such, the human response to immunization with the 17D line of vaccines has been used to identify gene signatures that correlate with desirable vaccine traits like immunogenicity [8], with the prospect of improving the designs of other vaccines.

Neutralizing antibodies elicited by the 17D-based vaccines have long been considered a correlate of protection against YFV. Titers are detected in humans as early as six days post vaccination and have been recovered after forty years [9,10]. Antisera protects non-human primates (NHPs; [11]) and intracranially (i.c.) inoculated mice [11,12] against challenge. Neutralizing antibodies rise to maximum levels around 2 weeks post-vaccination [13]. There is robust activation of CD4+ and CD8+ T cell responses that appear to transition into stable, polyfunctional memory [14–17]. Virus-specific CD4+ (conventional and T regulatory) and CD8+ T cells peak between 10 [17] and 11–30 [16,18,19] days post-vaccination, respectively. The magnitude of the CD8+ T cell response is influenced by viral load, peaking shortly after virus in the blood falls below detectable levels [19]. CD8+ T cells degranulate and produce IFNγ, IL-2, and TNFα [16,17]. CD4+ T cells predominantly produce IFNγ and express CXCR3, indicating a largely T_H_1 polarization [20]. Circumstantially, T cells appear to be capable of killing virus-infected cells [17]. Although it is widely speculated that YFV-specific T cells contribute to the superior efficacy of the 17D line of vaccines [21], this remains unproven as such questions cannot be addressed in human subjects.

Progress toward understanding, optimizing and exploiting 17D-based virus-elicited immunity has been hindered by the lack of a tractable small animal that accurately models severe YF disease and 17D-based vaccination. Few studies of naturally acquired wild type infections have examined immunologic parameters [22–24], and experimentally controlled studies in humans are virtually impossible. Therefore, human studies of the 17D line of vaccines have been limited to the evaluation of blood circulating factors [14–18,20,25,26]. NHP models have proven advantageous for studies modeling human disease and protection [11,27,28], but the cost of these studies is prohibitive. The resources and flexibility for immunologic studies in inbred rodent models are superior to NHPs. Unfortunately, normal and acquired immune-deficient rodents are refractory to infection and disease with both the 17D-based viruses and wild type YFV (wtYFV), with the exception of i.c. infection where both viruses are equally virulent [29,30].

Previously, we determined that subcutaneous (s.c.) infections with wtYFV were strongly restricted by the type I IFN response in mice [30], whereas in severe human or NHP infections, the virus appears relatively unhindered by the type I IFN system. Although the 17D-based viruses can elicit an immune response in immunocompetent mice [31,32], they are also restricted by type I IFNs making studies of immune development in these animals less relevant to humans where they actively replicate and spread from the site of inoculation. In mice lacking the receptor to type I interferon (IFNAR^-/-^), the 17D-204 vaccine strain replicates and disseminates from the site of s.c. inoculation, which functionally mimics human vaccination. 17D-204 is cleared in all mice following only minor clinical signs of infection. In contrast to 17D-204, infection with the wild type Asibi virus reproduces remarkably human-like disease [30], accompanied by a substantial cytokine response with high levels of IL-6 and MCP-I. This coincides with the viscerotropic dissemination of virus and severe liver and splenic pathologies similar to those found in post-mortem studies of YF patients [22–24,33]. Furthermore, wtYFV is uniformly lethal in these mice, reproducibly modeling the most severe aspects of YF disease.

Here, we demonstrate that vaccine substrain 17D-204 vaccinated C57BL/6 IFNAR^-/-^ (AB6) mice are completely protected against an otherwise lethal infection with wtYFV from 3 weeks to at least 1 year post-vaccination. Using this model, we have evaluated the formation of adaptive immunity to the 17D-204 virus in a context that closely resembles human vaccination, in that it is a self-limiting attenuated infection. Similar to human studies, 17D-204 infection of AB6 mice resulted in the induction of neutralizing antibodies and a large polyfunctional 17D-204-specific CD4+ and CD8+ T cell response. Passive and/or adoptive transfer of immunity into naïve mice implicated both humoral and cellular immunity for conferring protection against infection with wtYFV. 17D-204-specific CD4+ T cells conferred partial protection against challenge with wtYFV whereas complete protection was achieved only when anti-sera and CD4+ T cells were provided together. Virus-specific CD8+ T cells produced cytokines and were cytolytic but surprisingly offered no protective effect upon challenge. Our data provide evidence, for the first time, that some T cell subsets elicited by 17D-based vaccination can play a role in protection against wtYFV infection and disease, suggesting that T cells may contribute to the unparalleled efficacy of the 17D line of vaccines.

## Results

Previously [30], we demonstrated that s.c. footpad infection of IFNAR^-/-^ mice with wtYFV resulted in severe disease resembling human YF and requiring euthanasia. However, infection with the 17D-204 vaccine strain of YFV remained attenuated and resulted in clearance of the virus and only minor swelling at the site of injection. The clearance of 17D-204 suggested that infection was inducing an adaptive immune response capable of controlling virus infection. We therefore sought to define the cellular and humoral responses to 17D-204 as well as the protective efficacy against challenge with a virulent wtYFV.

The global success of the 17D line of vaccines resides in its ability to protect against challenge with diverse genotypes of wtYFV. Thus, we reasoned that a stringent test of protective immunity against wtYFV would require us to challenge with a genetically divergent strain of wtYFV. The 1971 isolate of YFV from Angola (Ang71, [34]) is a highly divergent phylogenetically distinct YFV virus of the Angola genotype [34–36]. Ang71 displays a 6.8 percent amino acid divergence from the 17D-204 parent strain, Asibi [36], making it an excellent wtYFV strain to assess the protective immunity elicited by the 17D-204 virus. Previously, we determined that Ang71 is virulent in IFNAR-/- mice [30].

**Fig 1:**
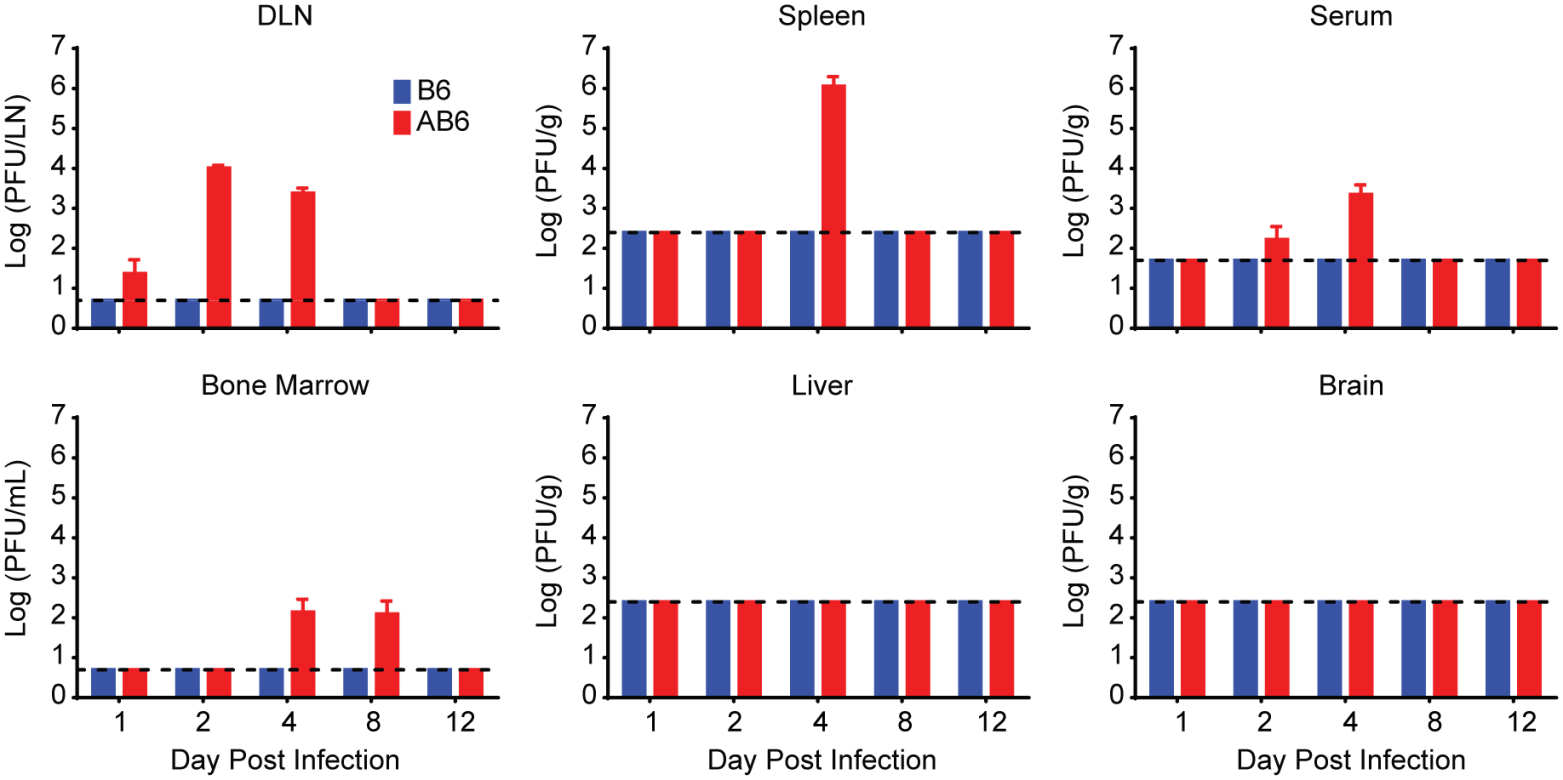
Replication and clearance of 17D-204 in AB6 mice. Five week old B6 or AB6 mice were infected s.c. with 1x10^4^ PFU of 17D-204 virus. On the indicated days p.i., mice were euthanized, perfused with virus diluent, and tissues were collected. Tissues were homogenized and a standard plaque assay was performed to determine the titers of 17D-204 in each tissue. The experiment was completed one time (B6, n = 6; AB6, n = 6–12).

**Fig 2:**
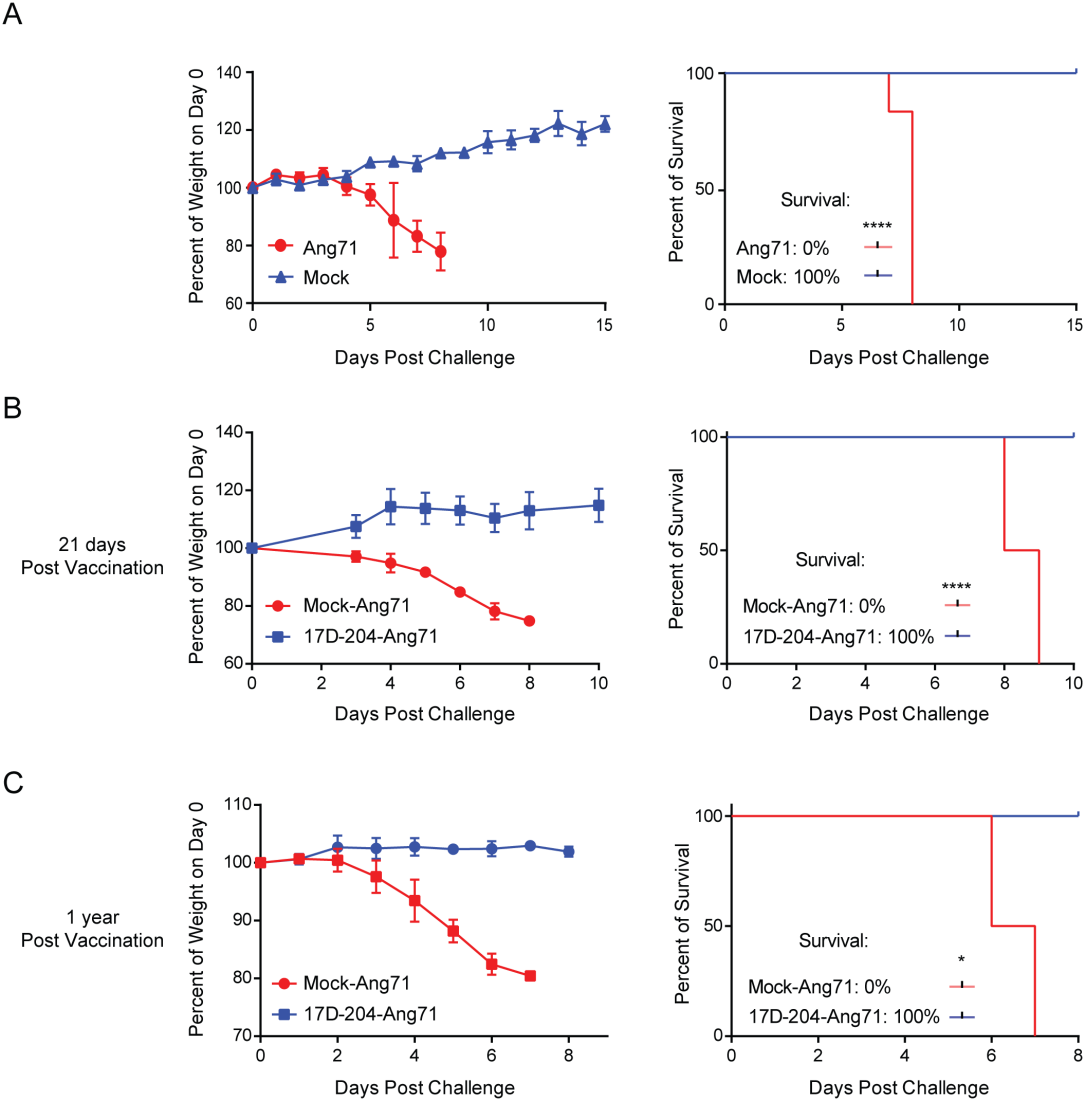
Immunization with 17D-204 protects mice against challenge with virulent wtYFV. (A) 5–6 week old mice were infected with Ang71 and monitored daily for weight and survival (mock, n = 5; Ang71, n = 10). (B and C) At 5 weeks of age, mice were infected with 17D-204 and on (B) d21 (Mock-Ang71, n = 7; 17D-204-Ang71, n = 12) or (C) 1 year (Mock-Ang71, n = 3; 17D-204-Ang71, n = 2) following vaccination, mice were challenged with Ang71 and monitored daily for weight and survival. Euthanasia was carried out according to preapproved weight loss and morbidity criteria. Weight is expressed as a percentage of weight on d0 of challenge with Ang71. The results in A and B are representative of two independent experiments. C was completed one time. Weights are displayed as mean ± SD. Statistical independence of surviving mice versus euthanized mice was calculated using a one-tailed chi-squared test (*, p ≤ 0.05; ****, p < 0.0001).

Our previous work was conducted in IFNAR^-/-^ mice from the 129 genetic background (A129) [30]. Virulence of the Asibi virus and Ang71 in A129 mice displayed an age-dependent attenuation that would make it impossible to complete studies of immune memory and protection when challenged with a virulent virus. We found that 17D-204 and Ang71 did not display the same age-dependent virulence in IFNAR^-/-^ mice on the C57BL/6 background (AB6). 17D-204 remained attenuated following infection of 5–6 week old AB6 mice with 1x10^4^ PFU per footpad, and resulted in only minor swelling at the site of injection. Similar to our previous observations in A129 animals [30], 17D-204 plaque forming units (PFU) remained undetectable in C57BL/6 mice whereas in AB6 mice, virus was detected in the serum, lymph nodes, spleen and bone marrow (Fig 1). Virus was never detected in the liver or the brain. 17D-204 was not detectable on d12 post infection (p.i.), suggesting that by this time the virus had been cleared from the AB6 mice. Importantly, subcutaneous injection of 2x10^4^ PFU per footpad of the Ang71 was found to be virulent in AB6 mice independent of age, including mice aged 5–6 weeks (Fig 2A), 8 weeks (Fig 2B), and one year (Fig 2C). Disease included progressive weight loss beginning on d4 p.i., swelling of the limbs surrounding the injection site, lethargy, piloerection, and hunched posture as described previously [30]. Ang71 was uniformly lethal in AB6 animals.

### Infection with the live-attenuated 17D-204 virus immunizes AB6 mice against challenge with a virulent, highly divergent strain of wtYFV

We hypothesized that immunity generated against 17D-204 in AB6 mice would be protective against infection with the virulent Ang71 strain of YFV. To test this, naive 5–6 week old AB6 mice were vaccinated s.c. with 1x10^4^ PFU of 17D-204 or virus diluent (mock) in both rear footpads. Mice were then challenged 21 days following vaccination by s.c. injections in both rear footpads with 2x10^4^ PFU of Ang71 (Fig 2B). The challenge with Ang71 in mice having received a mock vaccination resulted in disease and required 100% of animals to be euthanized by 9d p.i. In contrast, vaccination of mice with 17D-204 immunized them against challenge with Ang71 as evidenced by continued weight gain and the absence of clinical signs of disease. All 17D-204 immunized animals survived challenge (Fig 2B) with no clinical signs of disease for up to three months prior to being euthanized. Furthermore, AB6 mice having received a single 17D-204 immunization at five to six weeks of age were completely protected against clinical signs of infection when challenged one year later (Fig 2C). Thus, immunization with the live-attenuated 17D-204 YFV vaccine strain resulted in long-term protective immunity against challenge with a highly virulent, genetically divergent isolate of wtYFV.

**Fig 3:**
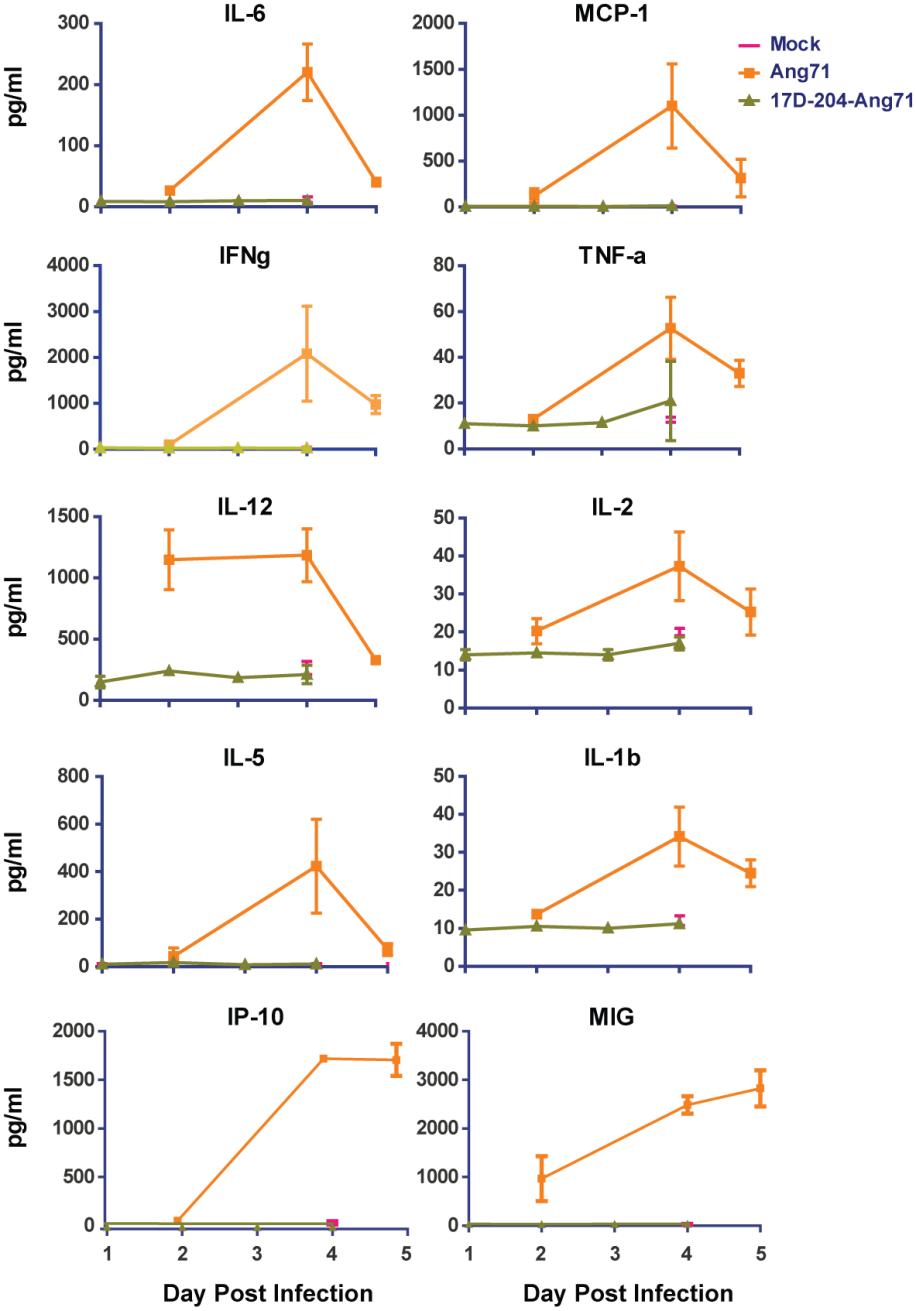
Immunization with 17D-204 protects mice against cytokine storm associated with virulent wtYFV infection. 5–6 week old mice received 17D-204 immunization or mock vaccination. Mice in the groups receiving Ang71 were infected on d21 after 17D-204 immunization, at approximately 8–9 weeks of age. On the indicated days p.i., serum was harvested and cytokine levels were determined by multiplex Luminex bead assay. Mock groups were collected at a 4d p.i. only. Samples for the Ang71 group were collected on d2, d4, and d5 p.i. Samples for the 17D-204-Ang71 group were collected on d1, d2, d3, and d4 p.i. Samples were collected from three separate experiments. Displayed data are n = 5 for mock, n = 3 for the other groups.

**Fig 4:**
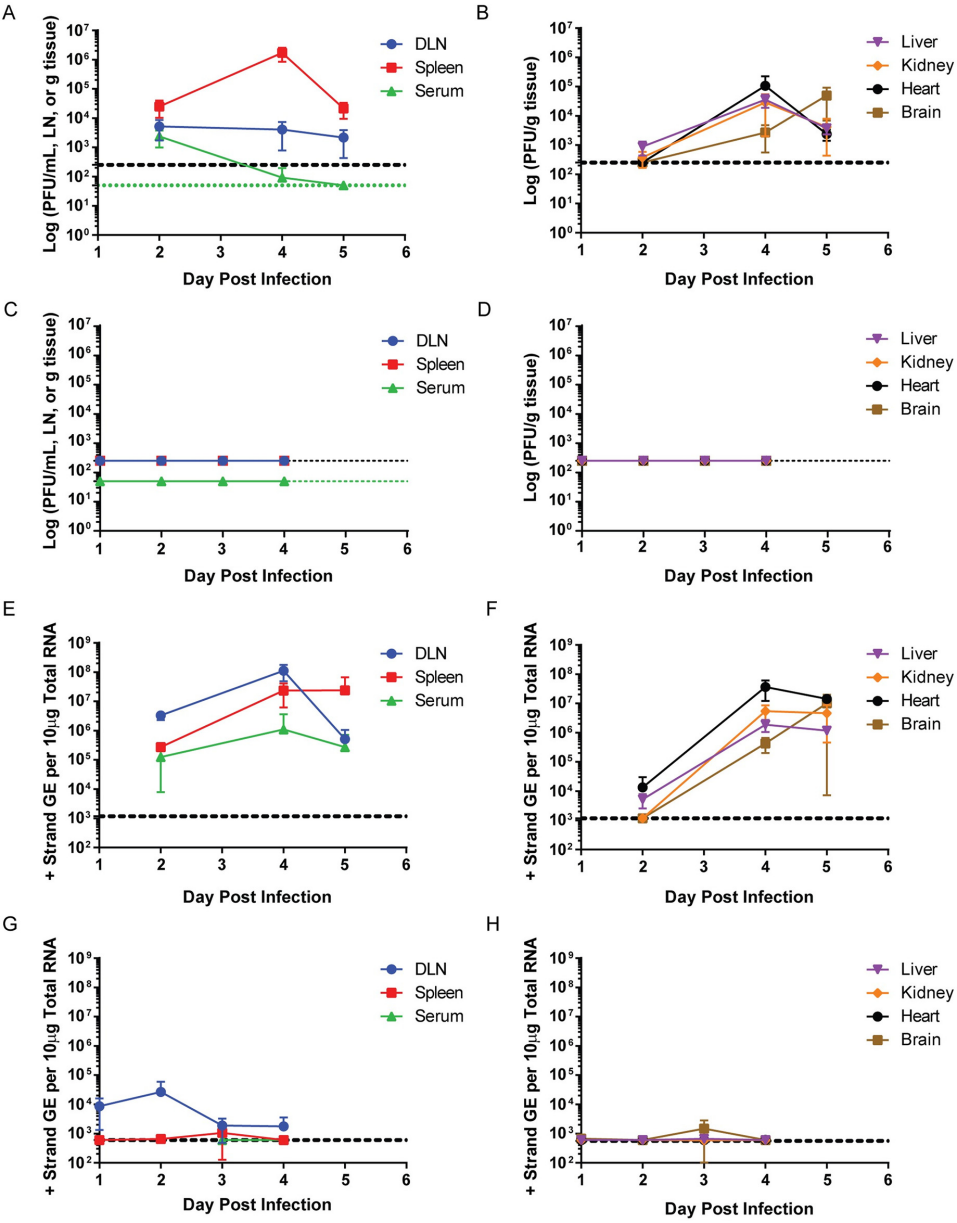
Immunization with 17D-204 restricts replication of wtYFV following challenge. (A-B, E-F) Naive or (C-D, G-H) 17D-204-vaccinated 8 week old mice were infected s.c. with Ang71. On the indicated days p.i., tissues were harvested and (A-D) PFU or (E-H) genomic equivalents [GE] were determined by (A-D) standard plaque assay or (E-H) RT-qPCR (n = 6 for all groups). The black hashed line indicates the (A-D) limit of detection [LD] or (E-H) limit of quantification [LOQ]. (A, C) The green hashed line indicates the LD for serum samples.

We previously reported that YFV infection of mice resulted in rapid and transient induction of high levels of the proinflammatory cytokines IL-6 and MCP-1 in serum [30], consistent with human studies of YF [22], YF vaccine-associated viscerotropic disease [37] and NHP infection with the wild type Asibi virus [IL-6 only, [38]]. We screened 18 additional serum cytokines in addition to IL-6 and MCP-1 (Fig 3) in mice following Ang71 infection and found that numerous cytokines with adaptive immunomodulatory roles were elevated including: proinflammatory and effector cytokines IFNγ and TNFα [39,40], IL-12—a proinflammatory T cell and IFNγ stimulating protein [41], IL-2 –a T cell growth factor [42], and the B-cell differentiation and growth factor IL-5[43]. All reached peak levels around 4d p.i., declining by d5. IL-12p70 levels were high on d2 through d4 p.i. before declining by d5. The immune cell chemoattractant proteins IP-10 and MIG peaked on d4 p.i. and remained at similarly high levels through 5 d p.i. Several additional cytokines were found to be largely non-responsive to Ang71 infection in non-immunized animals (S1 Fig). The peak of the cytokine storm coincided with the peak of viral titers in most tissues (Fig 4A and 4B), the onset of weight loss (Fig 2B) and the rapid progression of disease, suggesting that a broad cytokine response may be a biomarker for and a contributing factor to disease. Importantly, cytokine levels were not elevated in 17D-204-immunized animals, remaining similar to those recorded for mock-infected mice at d4 post challenge (Fig 3).

### 17D-204 immunization dramatically restricts wtYFV replication and dissemination upon challenge

The lack of disease observed in 17D-204-immunized mice when challenged with a lethal dose of Ang71 suggested the induction of a rapid and long-lived adaptive immune response that was efficient at controlling infection and/or dissemination of Ang71. To determine the degree to which replication and dissemination of Ang71 was restricted in 17D-204 immunized mice, we infected mice with Ang71 and harvested tissues at 2, 4 and 5 d post-challenge. In mock-vaccinated mice, infectious Ang71 was detected in the popliteal lymph node draining the inoculation site (DLN), serum and spleen within 2d (Fig 4A). DLN titers reached a sustained 2-5x10^3^ PFU/LN. Serum viremia peaked on d2 p.i. at ~2 x 10^3^ PFU/ml and fell below the LD in all but one animal on d4 and then in all animals by d5. Virus was detected in the liver in all but one mouse by 2d p.i. and in all mice by d4 and d5 p.i. Two mice had detectable virus in the kidney at 2d p.i. and all mice had virus in the kidney on d4 and d5 p.i. By d4 and d5 p.i. the heart and brain in all mice had detectable virus (Fig 4B). Generally, viral titers declined in the visceral tissues between d4 and d5. In the brain, the titer rose by approximately 20-fold. Despite rising levels of virus in the brain, as previously reported [30], the mice did not present signs of neurologic disease.

No tissues harvested from mice immunized with 17D-204 and then challenged with Ang71 contained any detectable infectious virus above the limit of detection (Fig 4C and 4D). For greater sensitivity, we compared viral GE by RT-qPCR in mock-vaccinated mice (Fig 4E and 4F) versus 17D-204-immunized mice (Fig 4G and 4H) after Ang71 challenge. RNA genomes for Ang71 were readily detected in unvaccinated AB6 mice. GE in the DLN and serum peaked on d4 p.i. and by d5 p.i. had decreased (Fig 4E). GE in the spleen increased through d4 p.i. and remained at similar levels through d5 p.i. GE in the liver, kidney, and heart remained similar from d4 to d5, whereas an increase in GE was observed in the brain from d4 to d5 (Fig 4F). In 17D-204-immunized mice, however, Ang71 RNA was consistently detected only in the DLN (Fig 4G) but at more than 100-fold lower levels compared to mock-vaccinated mice (Fig 4E) at d2 p.i. and approximately 6 x10^4^-fold lower by 4d p.i. In a subset of mice, the spleen [33%], liver [17%], kidney [17%] and brain [33%] showed low GE levels peaking at d3 p.i. and falling below the LOQ at 4d p.i. One mouse had low levels of detectable GE in the heart at 4d p.i. (Fig 4H). The variation in GE to PFU ratio observed across tissues and time points likely reflects the cellular dynamics and formation of adaptive immune responses, primarily neutralizing antibodies, against Ang71 that obscure the detection of PFU but not GE.

### 17D-204 convalescent serum imparts partial protection against challenge

**Fig 5:**
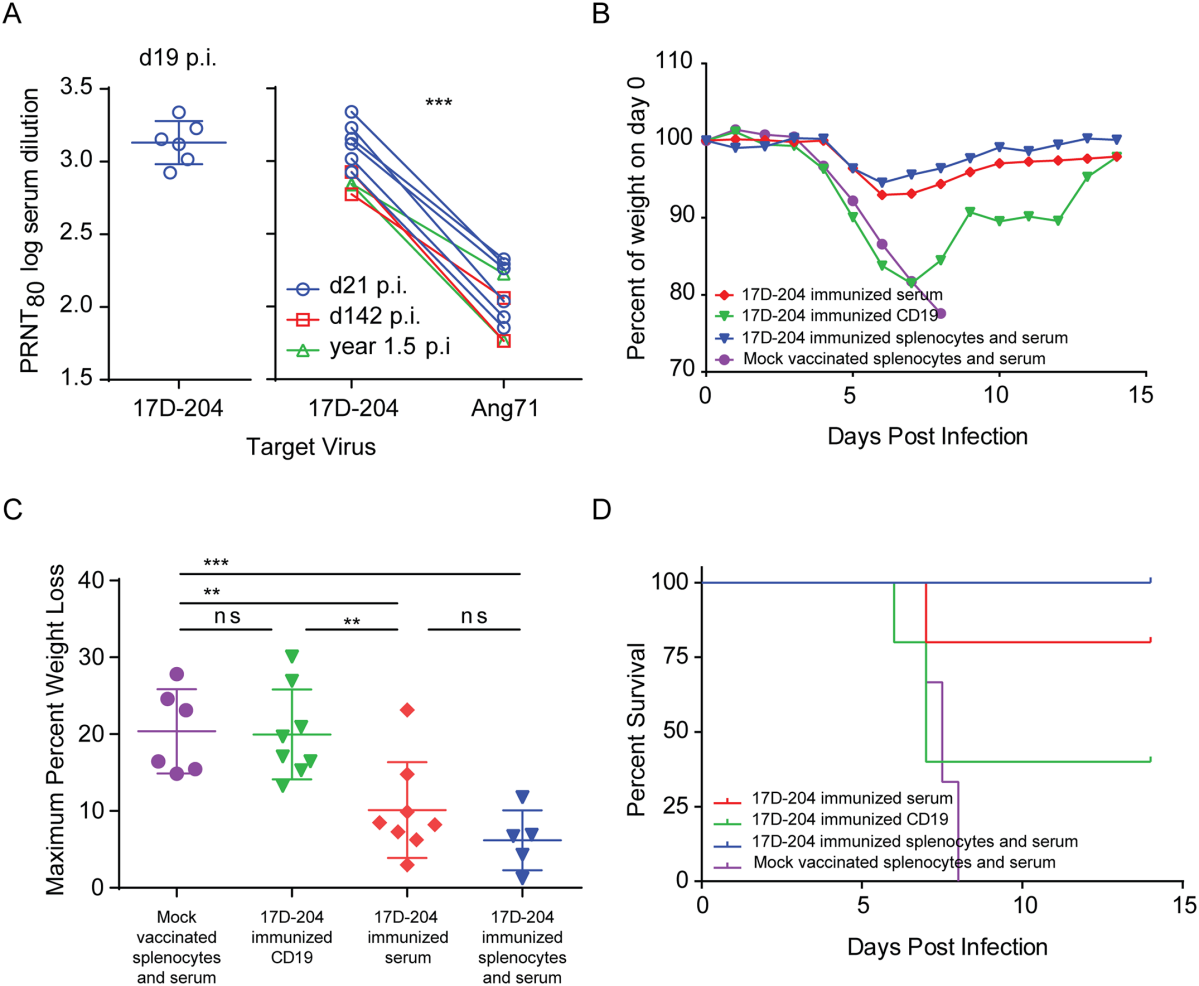
Transfer of convalescent serum or B cells from 17D-204 immunized mice to naïve mice partially protects against challenge with wtYFV. 5–6 week old mice were immunized s.c. with 17D-204. (A) serum was harvested and screened by PRNT_80_ for 17D-204 and Ang71 neutralizing antibody at the indicated days post immunization [***, p = 0.0001 paired t-test]. (B-D) On d21 following immunization, spleens, lymph nodes and sera were collected. CD19+ cells were enriched according to S2 Fig and adoptively transferred into naive 8 week old mice. Some mice received passive transfer of sera and total splenocytes from 17D-204 or mock vaccinated animals. Twenty-four hours after the transfer, mice were challenged with Ang71 and monitored daily for (B) cumulative weight loss (C) maximum weight loss and (D) survival. Weight is expressed as a percentage of weight on d0 of challenge [ns, not significant; **, p≤0.005; ***, p≤0.0005; One-way ANOVA with Tukey's multiple comparisons test]. Refer to Table 1 for statistics comparing percent of survival and numbers of animals. These data are the combined results of two independent experiments.

Sera collected from 17D-204-immunized mice were evaluated for 17D-204 neutralizing antibodies by plaque reduction neutralization test (PRNT; Fig 5A). Consistent with the high seroconversion rate in human vaccinees of between 90% and 100% [44,45], all mice tested between 19 days post immunization and 1.5 years post immunization demonstrated PRNT_80_ neutralizing antibody titers against both 17D-204 and Ang71 (Fig 5A). We found that serum neutralizing titers were reduced by approximately nine-fold on average against Ang71 when compared with 17D-204 virus. The reduced titers were most likely explained by the amino acid divergence displayed between 17D-204 and Ang71, as discussed above.

**Table 1:** Ang71 challenge following adoptive transfer of cells/sera from 17D-204-vaccinated mice into naive mice.

	Survival Outcome				Average Survival Time (AST)	
Adoptive Transfer	# Alive	# Dead	Percent Survival	P-value^a^	Days ± SD	P-value^b^
**Mock-Vaccinated Total Splenocytes +Serum**	0	6	0.0	n/a	7.58±0.49	n/a
**17D-204 Immunized CD4**	5	3	62.5	**	6.33±0.58	*
**17D-204 Immunized CD8**	0	8	0.0	ns	7.50±0.53	ns
**17D-204 Immunized CD19**	3	5	37.5	*	7.00±0.71	ns
**17D-204 Immunized Serum**	6	2	75.0	**	7.50±0.71	ns
**17D-204 Immunized CD4, CD8**	4	4	50.0	*	6.75±0.29	ns
**17D-204 Immunized CD4, CD8, Serum**	8	0	100.0	***	n/a	n/a
**17D-204 Immunized CD4, CD8, CD19, Serum**	7	0	100.0	***	n/a	n/a
**17D-204 Immunized Total Splenocytes + Serum**	5	0	100.0	***	n/a	n/a

^a^ p values were calculated using a one-tailed chi-squared test compared to mock. ns, not significant; *, p ≤ 0.05; **, p ≤ 0.005; ***, p ≤ 0.0005.

^b^ p values were calculated using a one-way ANOVA with Tukey's multiple comparisons test compared to mock. ns, not significant; *, p ≤ 0.05.

To test whether 17D-204 antisera would protect against challenge, sera and magnetically enriched CD19+ B-cells - those responsible for antibody production - (S2 Fig) were harvested from 17D-204 immunized mice on d21 after immunization and transferred into naive AB6 mice 24 hours before challenge with Ang71. To serve as positive and negative controls for protection, total splenocytes and sera from 17D-204-immunized or mock-vaccinated AB6 mice were transferred into naïve AB6 mice. Similar to the disease described in Fig 2, all mice [6/6] receiving total splenocytes and sera from mock vaccinated animals experienced weight loss (Fig 5B and 5C) and had to be euthanized (Fig 5D and Table 1). On average, animals receiving total splenocytes and sera from 17D-204-immunized mice experienced mild disease indicated by significantly reduced weight loss following challenge (Fig 5B and 5C) and 100 percent survival [5/5] (Fig 5D and Table 1). On average, mice receiving only serum from 17D-204 immunized animals also experienced mild disease as illustrated by significantly reduced weight loss compared to animals receiving mock-vaccinated splenocytes and serum (Fig 5B and 5C). Additionally, seventy-five percent [6/8] of mice receiving only serum survived challenge with Ang71, a significant increase compared to mice receiving mock splenocytes and serum (Fig 5D and Table 1). Mice receiving CD19+ cells from 17D-204 immunized mice experienced weight loss similar to mice receiving mock splenocytes and sera (Fig 5B and 5C). However, 37.5% [3/8] of mice receiving CD19+ cells from 17D-204 immunized mice survived challenge with Ang71, a significant increase compared to mice receiving mock serum and splenocytes (Fig 5D and Table 1). These data suggest that convalescent serum may be more effective than CD19+ cells at preventing severe disease. However, both serum and CD19+ B-cell recall responses can lead to survival of mice following challenge with Ang71.

### T cell immunity in 17D-204-immunized mice is robust

Acute viral infections, including the 17D-based vaccines, result in the proliferation of virus-specific CD4+ and CD8+ T cells responding to diverse epitopes. Once T cells become activated, they upregulate CD44 and downregulate CD62L (CD44_hi_CD62L_lo_) and represent circulating T cells. Although CD44 and CD62L comprise a conventional means of assessing T cell activation, CD11a is also upregulated on activated T cells in mice [46,47] and in humans [16]. Enhanced expression of CD11a on the surface of responding T cells is indicative of T cells responding to antigen-specific stimulus resulting from TCR cross-linking rather than bystander activation that can result from exposure to an inflammatory environment [46]. CD11a upregulation may constitute a more accurate means to assess total virus-specific T cell responses in systems where all possible T cell epitopes have not been defined [46]. Additionally, CD11a expression increases on YFV-specific CD8+ T cells in humans [16] and has been used to measure the broad T cell response to bacterial infection [46], malaria [48,49], and viruses [46,47] in mice. CD11a expression remains high for the life of a T cell following activation.

**Fig 6:**
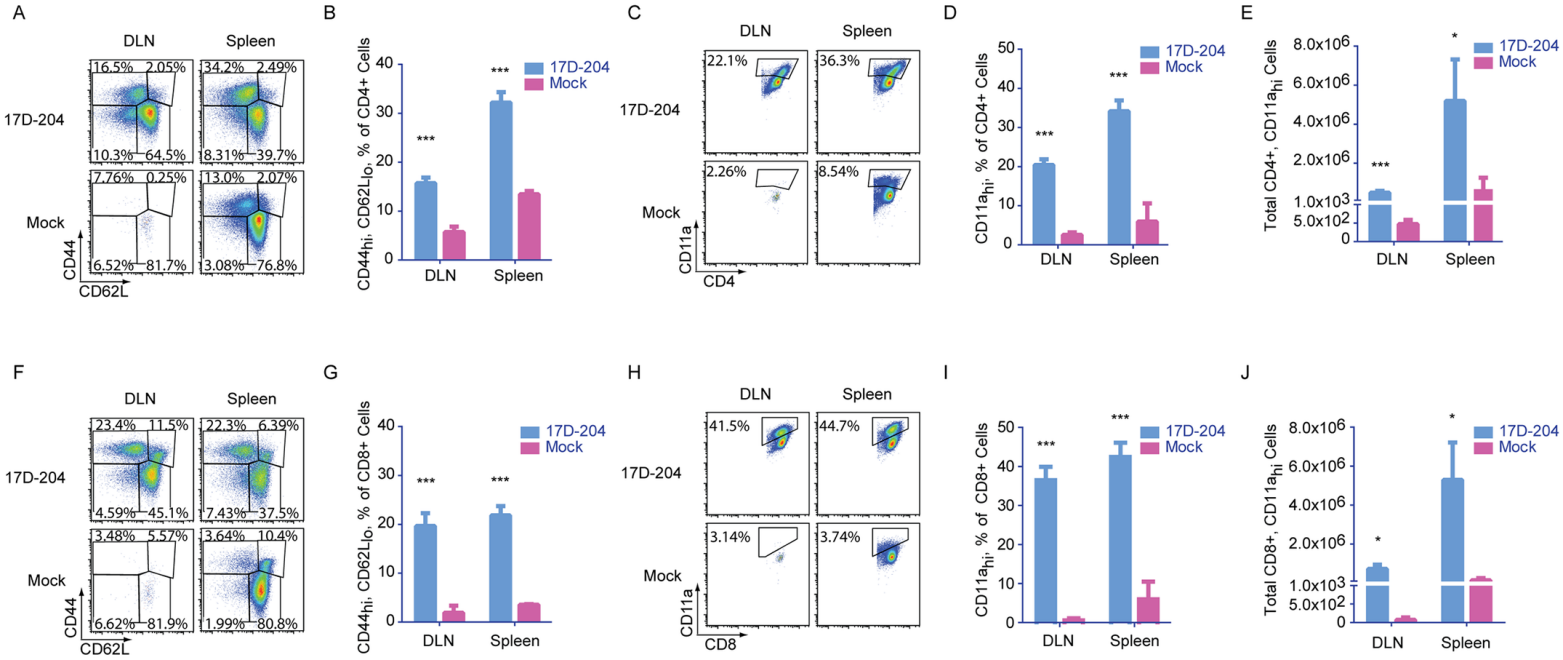
17D-204 immunization induces robust T cell responses. 5–6 week old mice were immunized with 17D-204. On d7 post immunization DLNs and spleens were harvested and (A-E) CD4+ and (F-J) CD8+ T cells were screened by flow cytometry for the expression of (A-B, F-G) CD44 and CD62L and (C-E, H-J) CD11a. Results are representative of two experiments (n = 3 mock, n = 6 17D-204). Data are displayed as mean ± SD. ***, p≤ 0.0001; *, p≤0.01 as determined by a Student's t-test).

To evaluate T cell immunity to 17D-204, we harvested popliteal DLNs and spleens from 5–6 week old immunized mice at d7 p.i. At this time, 17D-204 was not detectable in the lymphoid organs by plaque assay following the peak of replication on d4 p.i. (Fig 1). CD4+ (Fig 6A and 6B) and CD8+ (Fig 6F and 6G) T cells in 17D-204-immunized mice demonstrated an expansion of the CD44_hi_CD62L_lo_ subset commensurate with activation and proliferation. In addition, we evaluated T cells responding to 17D-204 by measuring CD11a upregulation. S3 Fig demonstrates the gating strategy for CD11a_hi_ cells and that the majority of CD11a_hi_ cells are CD44_hi_CD62L_lo_. Comparison of CD11a expression (Fig 6C, 6D, 6H and 6I) also demonstrated a significant increase in the percent of total CD4+ and CD8+ T cells over mock in the DLN and spleen. The increase in the percentage of activated cells corresponded to an increase in total numbers of CD11a_hi_ CD4+ and CD8+ cells indicating a true expansion of activated T cells responding to immunization with 17D-204 (Fig 6E and 6J).

**Fig 7:**
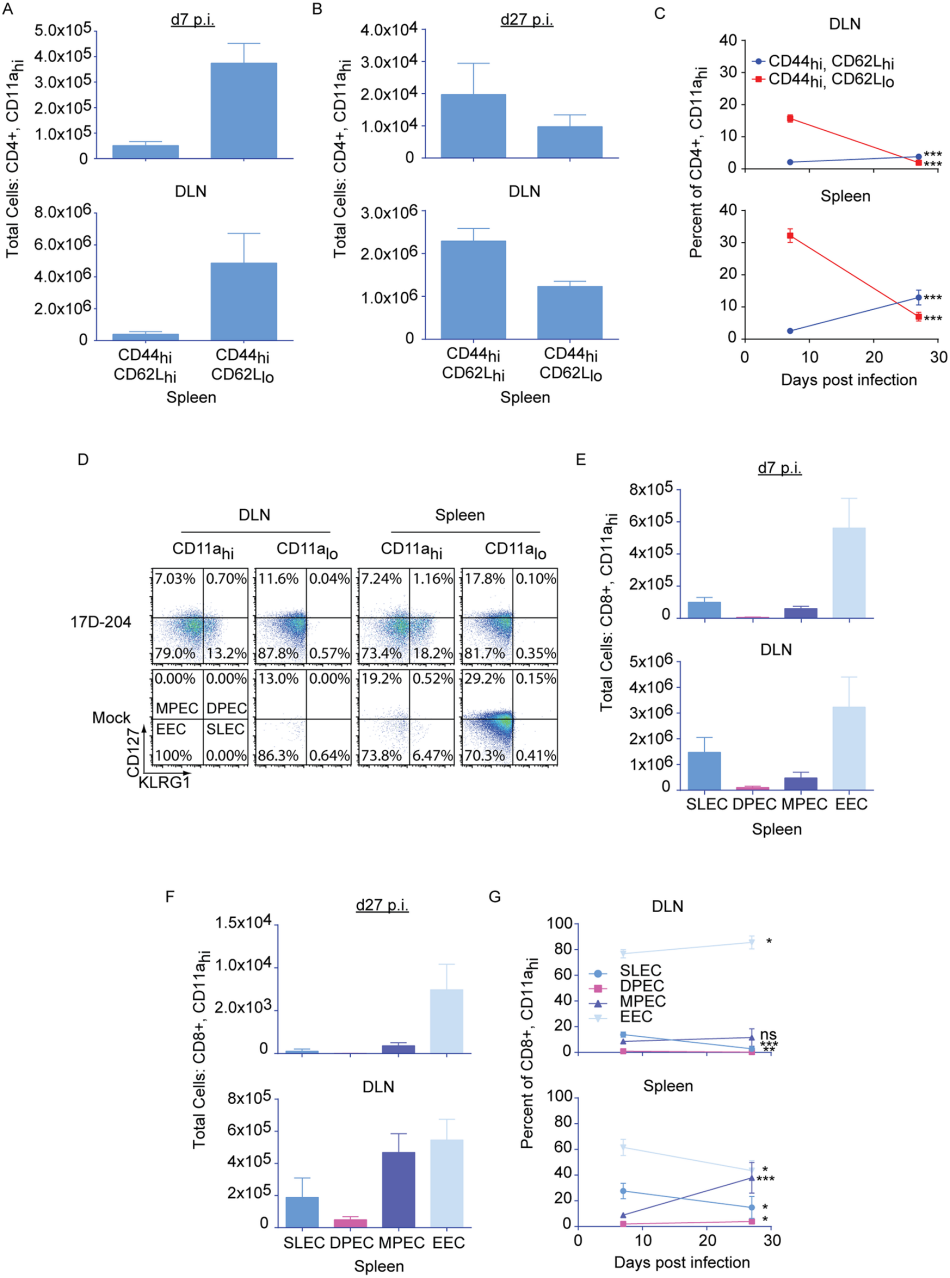
T cells responding to 17D-204 immunization express markers characteristic of the differentiation of long-lived memory. 5–6 week old mice were immunized with 17D-204. On d7 or d27 post immunization DLNs and spleens were harvested and cell surface expression of CD44 and CD62L was determined for (A-C) CD4+CD11a_hi_. (D-G) CD8+CD11a_hi_ cells were grouped as SLEC, DPEC, MPEC, or EEC by cell surface expression of KLRG1 and CD127. Results are from two separate experiments (n = 6 d7, 3 d27). Data are displayed as mean ± SD. ns, not significant; ***, p≤0.0005; **, p≤0.005; *, p≤0.05 as determined by a Student's t-test.

Following an acute T cell response to virus infection, T cells contract into memory, which can persist for the life of the animal. Memory cells can be broadly characterized as central (T_CM_, CD62L_hi_) and effector (T_EM_, CD62L_lo_) memory [50]. T_CM_ are characteristically found in the lymphoid compartments and are associated with the long-lived rapid recall responses that are most associated with adaptive immune memory [51–53]. T_EM_ are relatively short-lived cells that remain in circulation and retain effector functions similar to those seen in the acute response [51–53]. Compared to CD4+ T cells, differentiation of CD8+ effector T cell subsets have been more thoroughly defined by monitoring the expression of the inhibitory C-type lectin, KLRG1 and the IL-7 receptor-α, CD127 [54,55]. Four subgroups of effector cells have been described that predict the formation of memory (Fig 7D): early effector cells (KLRG1_lo_CD127_lo_, EEC) which probably represent an early state of transition into the other subtypes; short lived effector cells (KLRG1_hi_CD127_lo_, SLEC) which have a limited duration; memory precursor effector cells (KLRG_lo_CD127_hi_, MPEC) which give rise to long-lived memory and may be analogous to T_CM_; and double positive effector cells (KLRG1_hi_CD127_hi_, DPEC) which may be similar to T_EM_.

On d7 after 17D-204 immunization (acute T cell response) CD4+ T cells were predominantly CD44_hi_CD62L_lo_ (Fig 7A). Following contraction, at d27 after immunization, T_CM_-CD44_hi_CD62L_hi_ cells predominated in the DLN and the spleen (Fig 7B). As a proportion of total CD4+ T cells, T_CM_-CD44_hi_CD62L_hi_ cells were enriched from d7 to d27 post immunization. In contrast, the percent of total CD4+ T cells that were T_EM_-CD44_hi_CD62L_lo_ was decreased (Fig 7C). The acute CD8+ T cell response on d7 post immunization was predominated by EEC followed by SLEC (Fig 7D and 7E). A population of MPEC was present at d7 following immunization, suggesting that a subset of T cells was beginning to transition to long-lived memory. By d27 post immunization (Fig 7F), contraction of all cell types had taken place as indicated by the decrease in total numbers of all cell populations. The contraction corresponded to a decrease in the proportion of total CD8+ T cells of the SLEC and DPEC phenotypes in the DLN (Fig 7G). The EEC phenotype was enriched in the DLN on d27 post immunization, whereas the proportion of the MPEC phenotype remained unchanged. In contrast to the DLN, the contraction observed on d27 post immunization in the spleen corresponded to decreases in the proportions of SLEC and EEC. An increase in the proportions of the DPEC and MPEC phenotypes were observed in the spleen. The enrichment of MPEC cells in the spleen but not the DLN suggests that the formation of long-lived CD8+ T cell memory to 17D-204 may be dependent on the environments of specific lymphoid organs.

### 17D-204-specific T cells are polyfunctional

**Fig 8:**
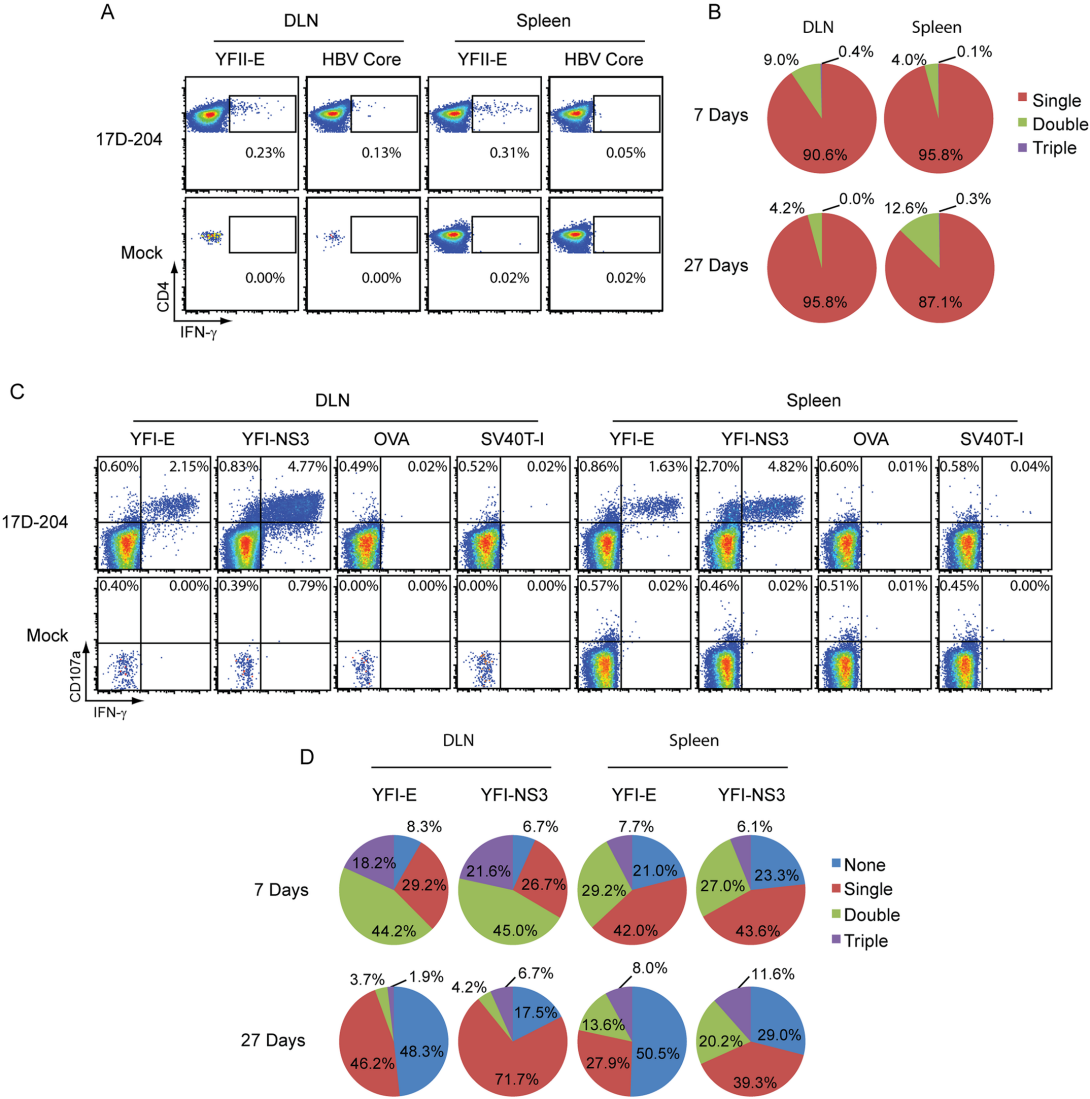
17D-204-specific T cells are polyfunctional. 5–6 week old mice were immunized with 17D-204. On d7 or d27 post immunization DLNs and spleens were harvested and the intracellular cytokine response was measured for (A-B) CD4+ T cells responding to *in-vitro* restimulation with the YFII-E peptide and (C-D) CD8+ T cells responding to *in-vitro* restimulation with YFI-E and YFI-NS3 peptides. Total CD4+ T cells were grouped by their ability to respond by producing multiple cytokines; (A-B) IFNγ, TNFα and IL-4 [Single, Double, or Triple]. CD8+,CD107a+ T cells (C-D) were grouped by their ability to produce IFNγ, TNFα and IL-2 [None, Single, Double, or Triple]. The results are representative of two experiments (n = 3 mock, n = 6 17D-204). A and C are representative dot plots. Percentages in A and C are percent of total CD4+ (A) or total CD8+ (C) cells. B and D display the mean percentages across all samples.

To verify that 17D-204 elicited a specific T cell response and to determine whether those cells were functional, we evaluated DLN and spleen cells from 17D-204-immunized mice by intracellular cytokine staining (ICS). Cells were stimulated with the YFV MHC-II (YFII-E; I-A^b^) and two MHC-I (YFI-NS3; H2-K^b^ and YFI-E; H2-D^b^) restricted determinants [31]. Stimulation with the YFII-E peptide resulted in CD4+ T cells producing IFNγ (Fig 8A). Since polyfunctional 17D-based vaccine-specific T cells have been observed in humans, we evaluated the CD4+ T cells' ability to produce multiple cytokines (IFNγ, IL-2 and IL-4) and grouped them by how many cytokines they produced (single, double, triple). The majority of CD4+ T cells at d7 and d27 post immunization produced only one cytokine, dominated by IFNγ (Fig 8B). The remaining cells produced IFNγ and IL-2 with no IL-4 detected above background levels.

Stimulation of T cells with YFI-E or YFI-NS3 resulted in the majority of CD8+ T cell responders producing both IFNγ and CD107a (LAMP-1) (Fig 8C). CD107a is deposited at the cell surface during T cell degranulation and is linked with a T cell's ability to lyse targets [56], suggesting that CD8+T cells were largely functional and capable of killing target cells. When we assessed CD8+ T cells, we found that nearly all cells responding with cytokines also produced CD107a. We gated on CD107a+ cells and grouped them according to their ability to produce combinations (none, single, double, triple) of IFNγ, TNFα or IL-2 in response to peptide stimulation (Fig 8D). On d7 post immunization, CD8+ T cells responding to both YFV peptides demonstrated polyfunctional behavior with double and triple positive cells most predominant in the DLN while the spleen was comprised of mostly single and double cytokine producers. At d27 post immunization, cells from both tissues produced fewer cytokines. This effect was most dramatic from CD8+ T cells in the DLN and specifically those cells responding to the subdominant YFI-E epitope. The trend that T cell polyfunctionality becomes less diverse over time is consistent with the literature studying human vaccination with the 17D line of vaccines [17].

### 17D-204-specific T cells are cytolytic

**Fig 9:**
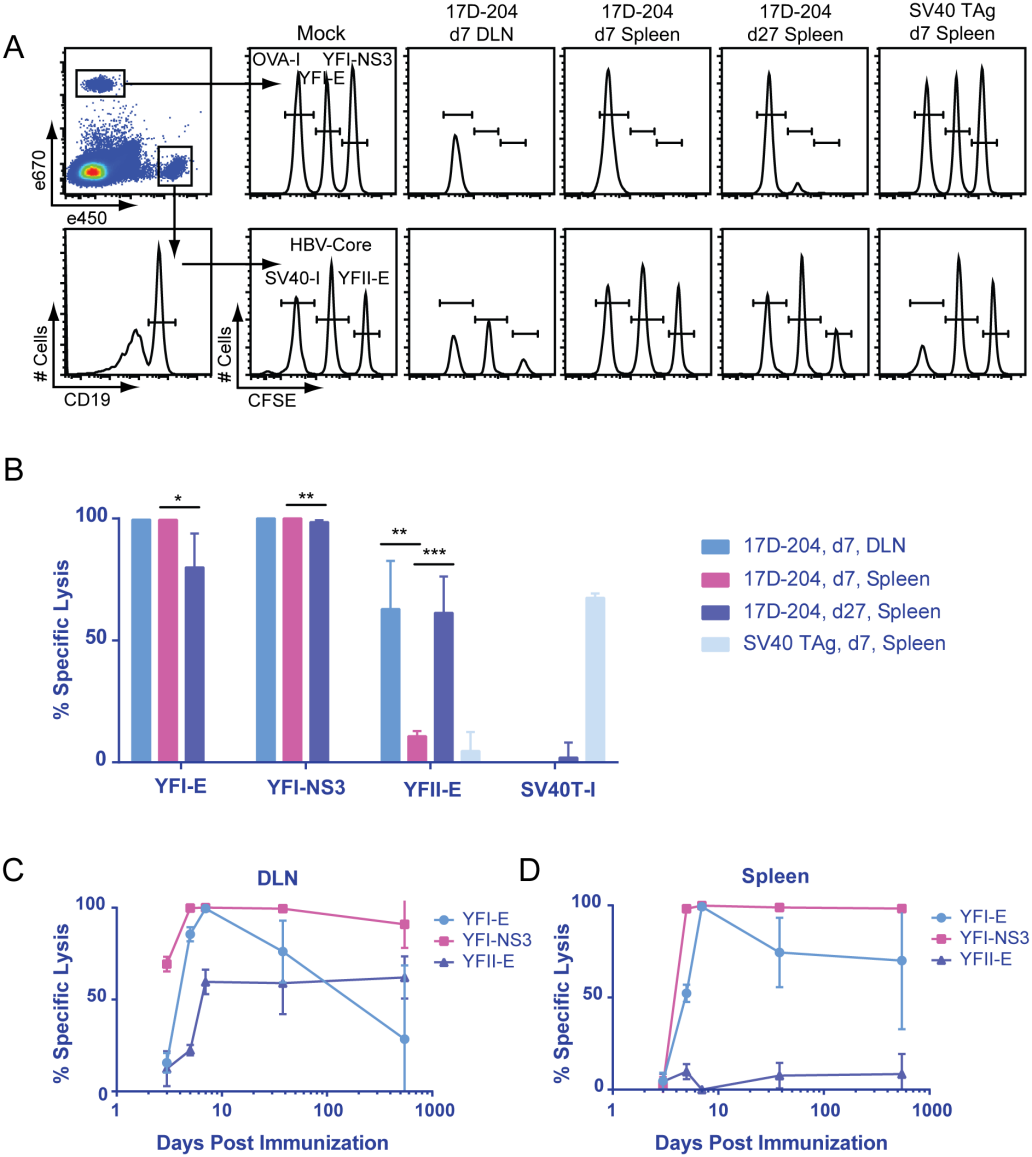
17D-204-specific T cells are cytolytic. 5–6 week old mice were immunized s.c. with 17D-204 or intraperitoneally with cells immortalized by the SV40 virus. Splenocytes from a naive mouse were loaded with the indicated peptides, fluorescently labeled, mixed, and adoptively transferred into the immunized mice. At the indicated times post vaccination, *in-vivo* cytotoxicity directed against splenocytes loaded with the indicated peptides was evaluated by (A) the displayed flow cytometric gating strategy. (B-D) Specific lysis was calculated as described in the methods. Results are representative of two experiments (n = 3–5 for d3-d38 p.i. and n = 2 for d547). Data are displayed as mean ± SD for individual animals. ***, p<0.001; **, p<0.01; *, p<0.05 as determined by Student's t-test.

We tested whether 17D-204-specific T cells were cytolytic by examining *in-vivo* cytotoxicity against 17D-204 determinants. Mice were immunized with 17D-204 on d7, d27 or 1.5 years prior to intravenous transfer of fluorescently labeled naive splenocytes loaded with control peptides from ovalbumin (OVA-I; H2-K^b^), SV40 large T-antigen (SV40 TAg; SV40T-I; H2-D^b^), hepatitis B core (HBV-Core; I-A^b^) or YFV peptides YFI-NS3, YFI-E or YFII-E. Sixteen hours after transfer, spleens or popliteal draining lymph nodes were harvested and analyzed by flow cytometry to determine specific cytolytic activity (Fig 9A). As a positive control for specific cytotoxicity, mice were immunized with SV40 virus-transformed cells and cytolytic activity was confirmed against the SV40T-I determinant of SV40 TAg [57] (Fig 9A and 9B). During both the acute response (d7 post immunization) and the early memory response (d27 post immunization), strong cytolytic activity was detected against all CD8+ T cell determinants with a significant decrease in activity between d7 and d27 post immunization (Fig 9B). Although weaker than CD8+ T cell activity, cytotoxicity was detected against the CD4+ T cell determinant YFII-E during the same time frame. The activity was relatively strong in the DLN on d7, showing a significantly higher signal than in the spleen. By d27 post immunization, the signal had increased in the spleen (Fig 9B). Cytolytic activity was detected as early as d3 p.i. in the DLN for all determinants. In both the DLN and spleen, cytolytic activity was detected out to one and a half years following immunization (Fig 9C and 9D). Activity against the YFI-NS3 and the YFII-E peptides showed no drop from peak levels during this time whereas activity against the subdominant YFI-E peptide decreased over time in both organs.

### CD4+ T cells contribute to protection against challenge with wtYFV but CD8 + cells do not

**Fig 10:**
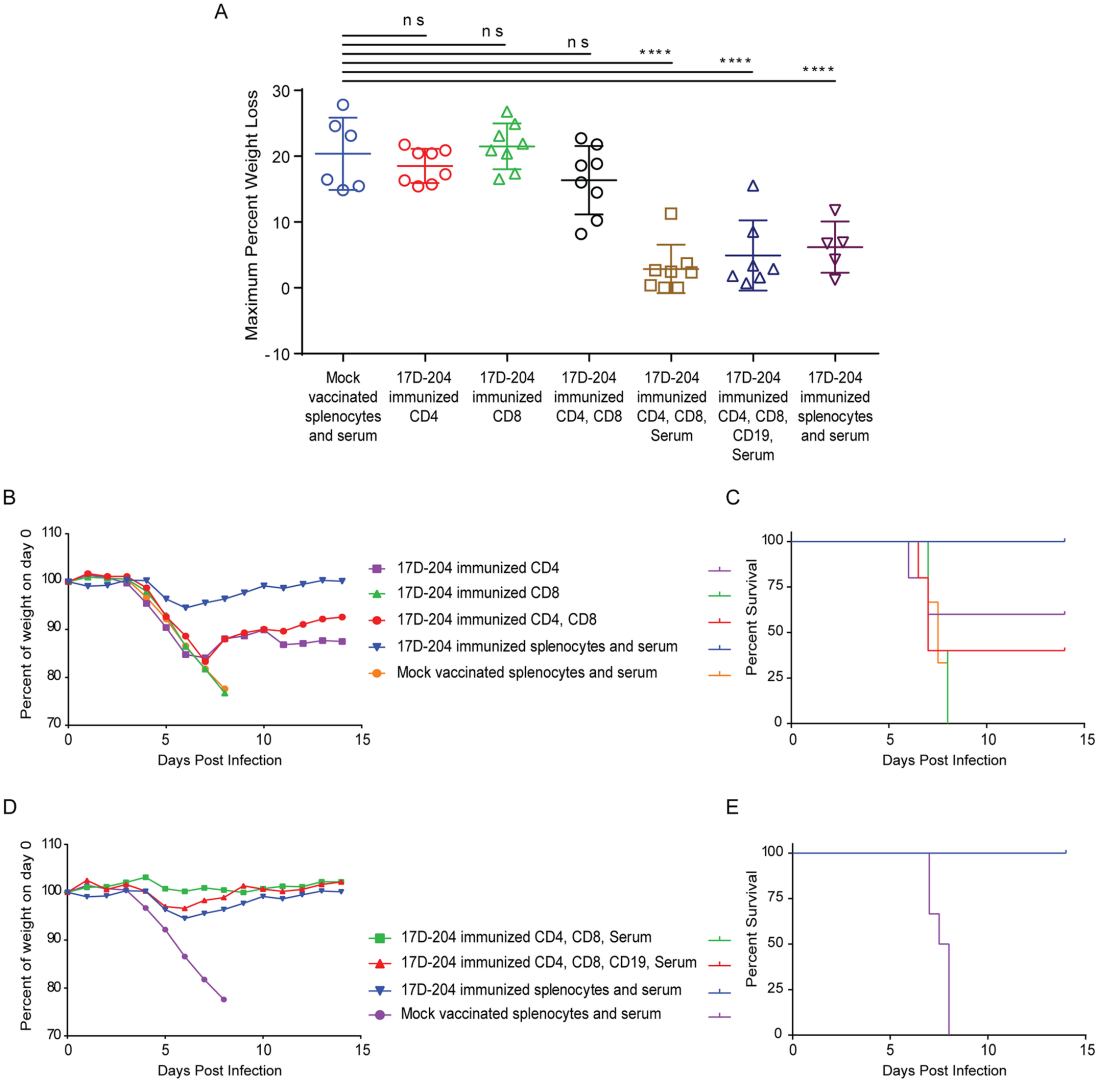
Complete protection against wtYFV requires serum and CD4+ T cells. Cell populations were magnetically enriched according to S2 Fig, mixed as indicated and adoptively transferred into naive eight week old mice. Some groups also received passive transfer of serum. Control groups received passive transfer of serum and total splenocytes from 17D-204 immunized or mock vaccinated animals. Twenty-four hours after the transfer, mice were challenged with Ang71 and monitored daily for (A -B, D) weight and (C and E) survival. Weight is expressed as a percentage of weight on d0 of challenge [ns, not significant; ***, p≤0.0005; One-way ANOVA with Tukey's multiple comparisons test]. Refer to Table 1 for statistics comparing percent of survival and numbers of animals. These data are the combined results of two independent experiments. These results were acquired simultaneously with those depicted in Fig 5, and control groups are shared with those depicted in Fig 5.

To determine whether T cells could contribute to protection against challenge with Ang71, we isolated serum and magnetically enriched CD4+, CD8+ or CD19+ cells (S2 Fig) on d21 post immunization and adoptively transferred combinations of the enriched populations into naïve AB6 mice. Mice were challenged with Ang71 and monitored for weight loss (Fig 10A, 10B and 10D and Table 1) and survival (Fig 10C and 10E and Table 1). Compared to mice receiving splenocytes and serum from mock vaccinated animals, mice receiving CD4+ T cells alone or in combination with CD8+ T cells experienced similar disease as indicated by weight loss. However, survival of both groups (CD4, [5/8]; CD4, CD8, [4/8]) was increased compared to mice receiving splenocytes and serum from mock animals. Surprisingly, mice receiving only CD8+ T cells from 17D-204 immunized mice experienced similar disease and mortality as indicated by weight loss (Fig 10A and 10B) and survival [0/8] (Fig 10C and Table 1) as animals receiving mock splenocytes and serum. Interestingly, the subset of animals that had to be euthanized after receiving CD4+ T cells from 17D-204 immunized mice experienced a significantly reduced AST, by approximately one day, compared to mice receiving mock vaccinated total splenocytes and serum (Table 1). Although overall the severity of clinical disease in this subset of mice was similar when comparing weight loss (Fig 10A and 10B) to what was observed in mice receiving mock-vaccinated splenocytes, the onset was earlier. This observation suggests that under as yet undefined conditions, CD4+ T cells may contribute to immunopathology following challenge with a wtYFV. Overall, these results suggest that in AB6 mice, 17D-204-elicited CD4+ T cells can impart protection against challenge with a wtYFV whereas CD8+ T cells are not protective.

### Humoral and cell mediated immunity act in tandem to confer protection against wtYFV

Since neither 17D-204 sera (Fig 5B–5D) nor T cells (Fig 10A–10C) alone were capable of completely protecting mice against Ang71 (Table 1), we adoptively transferred combinations of T cells, B cells and sera into naïve mice prior to challenge. When serum from 17D-204-immunized mice was transferred in addition to both CD8+ and CD4+ T cells, 100% of mice were protected against challenge with Ang71 compared to mice receiving total splenocytes and serum from mock vaccinated animals. These groups (CD8, CD4, Serum and CD4, CD8, CD19, Serum) displayed reduced weight loss (Fig 10A and 10D) and increased survival ([8/8] and [7/7]) compared to animals receiving splenocytes and serum from mock vaccinated mice. Since CD8+ T cells did not contribute to protection (Fig 10A–10C), these data suggest that CD4+T cells and sera from 17D-204 immunized mice can act together to confer protection against challenge with a wtYFV.

## Discussion

The success of the live-attenuated 17D line of YFV vaccines stems from the induction of long-lived, probably life-long, immunity against wtYFV. Due to the wide use of the 17D-based vaccines, volunteer vaccinees have in recent years contributed immensely to our understanding of vaccine-induced immunity in humans. These studies have shown that a single vaccination results in long-lived neutralizing antibodies [9,10] as well as long-lived [14,15] functional memory [16,17] following a robust CD4+ and CD8+ T cell response. Additionally, the use of systems biology approaches to track genomic signatures following immunization paints a picture that more thoroughly decodes the superior immunogenicity of the 17D line of vaccines and may lay groundwork for predicting the efficacy of other vaccines [8]. Despite these advances, studies linking humoral and T cell responses to protection against wtYFV have been lacking due to the absence of a cost-effective model that accurately recapitulates both vaccination and disease. In this study, we used an AB6 murine model to evaluate the immunity induced by 17D-204 immunization and its corresponding protection against challenge with a virulent wtYFV. Our results suggest that both neutralizing antibodies and CD4+ T cells elicited by 17D-204 can be protective against challenge with a virulent wtYFV.

Infection of AB6 mice with wtYFV elicits a broad elevation in the cytokine profile reminiscent of natural wtYFV infection in humans or rare vaccine-associated viscerotropic disease (YFV-AVD) [22,37,58]. Elevation of MCP-1, IL-6 ([30] and this study), TNF-a and IP-10 in the AB6 model is consistent with studied cases of patients that develop a fatal case of YF [22] or YFV-AVD [37,58]. The role of these and other elevated cytokines following wtYFV infection and YFV-AVD is unknown. However, pathological cytokine responses including IFNγ, IL-6, IL-10, TNFα, and IP-10 [59], all of which are elevated in AB6 mice during fatal Ang71 infection, have been implicated in inducing vascular permeability with the onset of hemorrhagic fever after infection with the related Dengue virus (DENV). Incidentally, serum cytokine concentrations become elevated on d4 p.i. ([30] and this study), marking the onset of clinical disease in AB6 mice. 17D-204-immunized AB6 mice show no signs of cytokine elevation following challenge with Ang71 possibly due to limited viral replication, which is mostly restricted to the DLN. We suggest that the cytokine response associated with high viral replication in unimmunized mice may contribute to disease. Future studies are warranted to assess whether suppressing these responses after Ang71 challenge of unvaccinated mice could prevent the onset of disease and perhaps lead to treatments for human YF.

The adaptive immunity induced by the 17D line of vaccines in humans has been partially characterized in recent years [14–18,26,60]. However, little has been studied concerning the specific components of this response that are required for protection against wtYFV. Antibodies are a correlate of protection against wtYFV and are considered by the WHO as the primary measure of vaccine efficacy [61]. The importance of antibodies for protection against wtYFV is supported by reports that lower levels of YFV-specific antibodies correlate with severe and fatal cases of YF in contrast to mild non-fatal cases [22]. Additionally, lethal infection in rhesus monkeys produces necrotic B cell germinal centers [28]. Our serum transfer results support the importance of antiserum for protection against wtYFV and suggest that it may be required for protection from disease. However, our results indicate that even in fully 17D-204-immune mice, Ang71 was not immediately sterilized upon challenge since virus was detected in the DLNs of all mice. It remains unknown whether 17D-based vaccines produce sterilizing immunity in humans against challenge with a wtYFV. Many experimental studies that have assessed the importance of antisera for protection have challenged with the Asibi virus, the parent virus to the 17D vaccine line. Our use of the divergent Ang71 virus, which is less efficiently neutralized in 17D-204-immunized mice, may explain why antiserum is not entirely protective against challenge. Long-term studies are needed to determine whether neutralizing antibodies alone are sufficient to clear virus or whether neutralizing antibodies simply limit the spread of virus while T cells or other immune cells eliminate virus reservoirs. Our findings support a critical role for neutralizing antibodies for protection against wtYFV.

As in humans, 17D-204 immunization of AB6 mice results in a robust expansion of polyfunctional CD8+ T cells that form functional memory. Our analysis shows that 17D-204-specific T cells produce IFNγ and a subset also produce combinations of TNFα and IL-2. Additionally, CD8+ T cells express surface CD107a following specific stimulation and efficiently lyse target cells *in-vivo* at early and late (memory) time points following vaccination. These observations led us to hypothesize that in this model the cytolytic capacity of CD8+ T cells would be important for controlling Ang71 in adoptive transfer experiments. A similar mechanism of control is seen in a related mouse model following vaccination against DENV [62] and West Nile virus (WNV) [63,64]. Instead, CD8+ T cells played no detectable role in protection against Ang71, as determined by the severity of disease or AST. Additionally, the fact that clinical disease and AST were not exacerbated also suggests that CD8+ T cells were not contributing to disease through immunopathologic mechanisms. The importance of CD8+ T cells for vaccine-related protection or challenge immunopathology in humans has never been tested.

We found that 17D-204-specific CD4+ T cells were functional during the early and late time points post immunization. 17D-204-specific CD4+ T cells displayed a strong T_H_1 polarization during the acute response, and similar cytokine profiles were maintained following the formation of memory. *In-vivo* cytolysis of targets displaying 17D-204 derived MHC-II restricted peptides was detected with similar efficiencies during both the acute and long-term memory response. Importantly, adoptively transferred CD4+ T cells contributed to survival following Ang71 challenge, resulting in the recovery of a majority of infected mice. These results suggest that CD4+ T cells may functionally contribute to the superior efficacy of the 17D line of vaccines and as such constitute a critical component for effective vaccination against wtYFV. The mechanism by which CD4+ T cells exert their effect remains unknown. However, the MHC-II restricted *in-vivo* cytotoxicity suggests that direct cytolysis by YFV-specific CD4+ T cells may be an important mechanism. Perforin and Fas/FasL mediated mechanisms, as measured by *in-vivo* cytotoxicity are involved in the control of WNV [65]. The same mechanisms that contribute to the protective efficacy of CD4+ T cells may also be involved in the CD4-mediated immunopathology that we observed in a subset of mice. Bystander cytotoxicity was observed from CD4+ T cells responding to DENV [66]. Determining the specific mechanisms by which CD4+ T cells promote their effects is outside the scope of this study but remains an important question for future studies. In order to understand the implicit requirements for control of wtYFV, it will be important to study the mechanisms that lead to CD4+ T cell-mediated protection and the apparent inability of CD8+ T cells to limit disease.

To conduct these studies, we have used a murine system that lacks type I interferon signaling. Type I interferon can act as a third signal [67] of T cell activation and is a pro-survival and proliferative cytokine [68] possibly affecting the responses in AB6 mice. Type I interferon plays a minimal role in T cell responses for some pathogens due to compensation by other third signal cytokines like IL-12 or as yet undefined pathogen-specific environments [68,69]. For example, Lymphocytic Choriomeningitis Virus (LCMV)-specific T cells lacking IFNAR have substantially inhibited proliferation whereas during *Listeria monocytogenes* (LM) infection, IL-12 acts as the predominant third signal [69–72]. The CD4+ and CD8+ T cell responses induced following 17D-204 vaccination were robust even in the absence of type I interferon signals, and were comparable in size to those seen against LCMV and LM in B6 mice [46,47]. Importantly, the size of the dominant CD4+ and CD8+ T cell responses in the IFNAR^-/-^ mouse model accurately reflects the response to individual dominant epitopes in humans [16,17]. These observations suggest that the magnitude of activated T cell responses in 17D-204 vaccinated IFNAR^-/-^ mice may accurately represent human responses.

The influence of type I interferon on the adaptive immune response in humans to vaccinations with a 17D-based vaccine is unknown. Numerous publications indicate that human cells produce type I interferon in response to a 17D-based virus infection [73–78]. It is unknown what influence type I interferon has on human T cells during vaccination with the 17D line of vaccines, thus we cannot rule out the existence of deficiencies in the T cell phenotypes elicited by IFNAR^-/-^ mice. To more carefully consider the role of type I interferon in humans, we assessed whether the 17D vaccine line induces a substantial type I interferon response in humans by independently analyzing gene expression data from PBMCs of human vaccinees published by Querec et al. [8]. This analysis indicated that immunization of humans with the 17D vaccine line does not result in significant increases in IFNα/β gene transcription in PBMCs (S4 Fig). Our analysis does not rule out more localized production of type I interferons (e.g. in the lymph nodes) or in a small subset of PBMCs, and the original study did not measure levels of protein in the sera. However, should these transcription data be representative of the levels of type I interferon following 17D-based vaccination, it suggests that type I interferons may play a limited role physiologically for 17D based vaccine-specific T cells. Under the same scenario, the proliferation of 17D-based vaccine-specific T cells in humans may be driven by an undetermined third signal cytokine, or T cell expansion may be driven by abundant antigen from relatively high titers of virus in the lymphoid compartments [30].

In summary, we have used a murine model of YFV infection and disease to characterize the immune response to 17D-204 and determine empirically which immune constituents contribute to protection against wtYFV. The 17D-based vaccine strains constitute one of the world's most successful vaccines, demonstrating superior safety, efficacy and longevity compared to most subunit vaccines and LAVs. The characteristics of immune development and function seen following 17D-204 immunization in small animal models can serve as benchmarks for the development of other vaccines that aim to mimic its superior immunogenicity and long-lasting protection. Since we can now assess the role that cellular immunity plays in conferring protection, we can form a more complete picture of what contributes to vaccine efficacy in general. More specifically, 17D-204-elicited protection against wtYFV, defined by neutralizing antibodies and CD4+ T cell immunity, may be directly applied towards the development of vaccines against other flaviviruses like DENV and WNV. Continued study of 17D-204-elicited immunity may facilitate vaccine research by uncovering novel immune mechanisms of action, discovering innate immune correlates of protection, developing immunotherapies, or providing a platform for screening vaccine candidates.

## Materials and Methods

### Ethics statement

Animals were maintained and procedures were performed in accordance with the recommendations in the Guide for the Care and Use of Laboratory Animals of the National Research Council. Protocols 1004668, 1103456, and 14033545 were approved by the University of Pittsburgh's IACUC committee. Approved euthanasia criteria were based on weight loss and morbidity.

### Mice, vaccination and challenge

Mice deficient in receptors for type I interferons, (IFNAR^-/-^, AB6) were bred under specific pathogen-free conditions. At 5–8 weeks of age, randomized male and female mice were transferred to the ABSL-2 or ABSL-3 facility for infection. Vaccination was administered subcutaneously (s.c.) in both rear footpads to 5–6 week old AB6 mice with 10^4^ PFU of 17D-204 in a volume of 10ul in an ABSL-2 facility. Mock-vaccinated animals received 10uL of virus diluent (PBS[with Mg+, Ca+] containing 1% donor calf serum). Mice were observed for clinical disease and weighed daily. At 8–9 weeks of age, mice were transferred to an ABSL-3 facility and challenged s.c. in both rear footpads with 2x10^4^ PFU Ang71 in a volume of 10uL. Mice were monitored daily for clinical disease and weight loss. Animals were euthanized based on morbidity or weight loss of 20%. For virus titers and qPCR, serum was separated from whole blood by centrifugation using microtainer tubes (BD). Mice were perfused with 10mL of virus diluent prior to tissues being collected and frozen at -80C in virus diluent or Tri Reagent-LS (MRC) until the time of processing. 50uL of serum was frozen undiluted for titer or placed in Tri Reagent-LS for qPCR.

### Cells

Vero (ATCC-CCL-81), Huh7 (Charles M. Rice, The Rockefeller University) and B6/WT-19 (Todd D. Schell, The Pennsylvania State University: College of Medicine) cells were maintained in Dulbecco's modified Eagle's medium (DMEM), supplemented with 10% fetal bovine serum (FBS), 0.29 mg/mL L-glutamine, 100 U/mL penicillin and 0.05 mg/mL streptomycin (37C; 5% CO2). B6/WT-19 cells are transformed by SV40 virus [79,80].

### Virus

Stocks of YFV 17D-204 were produced from infectious clone [81] by electroporation of *in-vitro* transcribed (IVT) viral genomic RNA. 1ug of infectious clone DNA was linearized by restriction digest with Xho1. Linear DNA was purified and used as a template for IVT (mMESSAGE mMACHINE SP6, Ambion). 20ug of IVT RNA was electroporated twice into vero cells during exponential growth phase harvested from three 50% confluent T175 tissue culture flasks using the following settings: 220V, 1uF, exponential decay. Electroporated cells were seeded into a single T175 flask in 15mL of media with HEPES (0.02M) and sodium bicarbonate (0.15%) and incubated for 7 days at 37C + 5% CO2. Supernatant was harvested by centrifugation at 4000 RPM for 30 minutes and stored at -80C. The Ang71 (14FA [34,35]) virus was amplified on vero cells as described previously [30]. Infectious virus titers were determined by a plaque assay on Huh7 cells, expressed as plaque forming units (PFU)/mL.

### Quantitative PCR

RNA was isolated first by crushing tissue in Tri Reagent-LS and following the protocol provided by the manufacturer. Polyacryl carrier was added for serum isolation only. Reverse transcription of 100ng of purified RNA was performed for +-strand viral RNA using primer T7-YFV-antisense (GCGTAATACGACTCACTATATACCATATTGACGCCCAGGGTTTT) targeting a region of the 5'-non-coding region that is conserved between Ang71 and 17D-204 and 18s-antisense (CGAACCTCCGACTTTCGTTCT) using TaqMan reverse transcription reagents (AB). Synthesis of cDNA consisted of 25C, 10 m followed by extension at 48C, 30 m and concluding with inactivation of RT at 95C, 5 m. Quantitative determination of YFV and 18s cDNA was performed using separate reactions in Maxima Probe qPCR Master Mix (Thermo) and read on a 7900HT Real-Time PCR System (AB). Primers for YFV: T7 (GCGTAATACGACTCACTATA), YFV-sense (AATCGAGTTGCTAGGCAATAAACAC), and YFV-Probe (CAGTTCTCTGCTAATCGCTCAACGAACG). Primers for 18s: 18s-antisense, 18s-sense (CGCCGCTAGAGGTGAAATTCT) and 18s-Probe (CAAGACGGACCAGAGCGAAAGCATTTG). Cycling conditions consisted of: denaturing and polymerase activation at 95C, 10 m; followed by 40 cycles of denaturing at 95C, 15 s then extension at 60C, 1 m. Fluorescence intensity data was collected during the extension step. The YFV GE standard curve was based on 10 fold dilutions of 17D-204 IVT RNA. The LOQ was set to the GE represented by the greatest dilution of standard remaining on the logarithmic curve. We found that expression of 18s in 100ng of RNA varied among tissues, thus mean 18s Ct values were calculated for each tissue type (e.g. spleen, brain, etc.) and these values were used to correct for sample loading error.

### Cytokine analysis

A mouse cytokine 20-plex panel kit was purchased from Invitrogen (LMC0006). According to the manufacturer's instructions, serum was diluted 1:1 with diluent and analyzed using a Luminex 100/200 instrument.

### PRNT80

Serial dilutions of control antibody or serum were incubated for 1 hour at 37C + 5% CO2 with approximately 100 PFU of 17D-204 or Ang71. PFU of non-neutralized virus was determined using a standard plaque assay on Huh7 cells. Plaques remaining at all dilutions were counted and expressed as a percent of plaques remaining with mock serum samples. A best fit non-linear curve constrained to maximum 100 percent of mock and minimum zero percent of mock was used to calculate the 80 percent reduction in PFU.

### Synthetic peptides

Peptides were ordered from GenScript at > 90% purity and resuspended in PBS + 5% DMSO at 1mM concentration. Peptides designated as YFI or YFII are respectively MHC-I or MHC-II restricted peptides originating in 17D-204. The designations E or NS3 indicate the 17D-204 protein in which the peptide originates. Peptides: YFI-E (4–12) IGITDRDFI; YFI-NS3 (268–275), ATLTYRML; YFII-E (231–245), LVEFEPPHAATIRVL [31]; SV40T-I (206–215), SAINNYAQKL [82]; OVA-I (255–262), SIINFEKL; HBV Core (128–140), TPPAYRPPNAPIL. All YFV peptides are conserved between 17D-204 and Ang71.

### ICS, flow cytometry and antibodies

ICS and cell surface staining was performed on single cell suspensions from spleens or lymph nodes created by pushing the tissues through a 70uM nylon mesh (Fisher). For ICS, up to 4x10^6^ total cells were incubated in 200uL of T cell growth media (RPMI 1640 containing 10% FCS, 100 U/mL penicillin, 20uM 2-ME, 1mM sodium pyruvate and 10mM HEPES) supplemented with 1uM of peptide, monensin (BD GolgiStop) according to the manufacturer's instructions and 1:50 anti-CD107a-PE. Cultures were incubated for 5 hours at 37C and 5% CO2.

Staining for ICS: Cell were stained for 30 minutes on ice with 1:1000 dilution of Invitrogen Blue LIVE/DEAD stain in PBS. Cells were washed 1x in FACS buffer (PBS supplemented with 2% FBS and 0.1% sodium azide). Cells were then incubated in a 1:200 dilution of rat anti-mouse CD16/CD32 on ice for 20 min then washed 1x with FACS buffer. Fluorophore conjugated antibodies (1:200 dilution) were added to the cells and incubated for 30 minutes on ice than washed 3x with FACS buffer. Cells were then fixed and permeabilized using Cytofix/Cytoperm solution (BD) on ice for 20 minutes then washed 3x in Perm/Wash (BD). Staining was completed with 1:200 dilutions of fluorophore conjugated antibodies in Perm/Wash for 15 min at room temperature then washed 3x with Perm/Wash. Cells were fixed in 4% PFA in PBS and stored at 4C O/N prior to analysis.

Surface staining: Cells were stained for 15 minutes at 1:1000 dilution of Invitrogen Blue LIVE/DEAD stain in PBS. Cells were washed 1x in FACS buffer then incubated in a 1:200 dilution of rat anti-mouse CD16/CD32 for 15 minutes then washed 1x with FACS buffer. Fluorophore conjugated antibodies (1:200 dilution) were added to the cells and incubated for 15 minutes at room temperature than washed 3x with FACS buffer prior to fixation in 4% PFA. FACS analysis was performed the following day.

All data were collected using a LSR II or Fortessa flow cytometer (BD) administered by the University of Pittsburgh’s Unified Flow Core. If available, a minimum of 300,000 live events were collected based on FSC-A/SSC-A and live/dead gating. Data were analyzed using FlowJo software (Tree Star). The following flow cytometry antibodies were purchased from eBiosciences or Tonbo biosciences: CD8α (53–6.7), CD4 (GK1.5), CD19 (eBio 1D3), CD44 (IM7), CD62L (MEL-14), CD11a (M17/4), IFNγ (XMG1.2), TNFa (MP6-XT22), IL-2 (JES6-5H4), IL-4 (11B11), KLRG1 (2F1), CD127 (A7R34), CD107a-PE (eBio1D4B).

### *In-vivo* cytotoxicity

Splenocytes from naive AB6 mice were incubated for 1 hour at 37°C in complete media with 1uM of the indicated peptides. Free peptide was removed by washing three times in PBS. Each splenocyte population was fluorescently marked by staining with 1uM eFluor 450 or eFluor 670 Cell Proliferation Dye (eBioscience) and 5, 0.5 or 0.05 uM CFSE (eBioscience) in PBS for 10 minutes at 37°C. Staining was halted by addition of T cell growth media then cells were washed 3 times in PBS, combined in equal ratios then transferred to infected mice. 16 hours following transfer, spleens and/or popliteal lymph nodes were harvested and stained with anti-CD19 prior to evaluation by flow cytometry. As a positive control for cytolytic T cell activity, a group of mice was immunized i.p. with 2x10^7^ B6/WT-19 cells and evaluated on d7 following immunization for specific lysis against the SV40 TAg site I determinant (SV40T-I). The number of splenocytes remaining in the MHC-I restricted peptide populations (YFI-NS3, YFI-E, OVA-I and SV40T-I) was calculated from total splenocytes. The number of cells remaining in the MHC-II restricted peptide populations (YFII-E and HBV-Core) was calculated using total CD19+ splenocytes. Specific lysis was calculated by the following formula for the YFI-NS3 peptide: ([Peptide in mock mice/OVA-I in mock mice]–[Peptide in immunized mice/OVA-I in immunized mice]) / [Peptide in mock mice/OVA-I in mock mice]. Specific lysis was calculated by the following formula for the YFI-E peptide: ([Peptide in mock mice/SV40T-I in mock mice]–[Peptide in immunized mice/SV40T-I in immunized mice]) / [Peptide in mock mice/SV40T-I in mock mice]. Specific lysis for the YFII-E peptide was calculated using: ([Peptide in mock mice/HBV-Core in mock mice]–[Peptide in immunized mice/HBV-Core in immunized mice]) / [Peptide in mock mice/HBV-Core in mock mice].

### Magnetic enrichment and adoptive transfer

Splenocytes and lymphocytes from 17D-204 immunized mice, on d21 post immunization, were pooled and stained with MicroBeads (Miltenyi) as per the manufacturer’s recommendations. Stained cells were subjected to positive selection using the AutoMACS automated sorting system. First the CD19 positive fraction was collected. Then the CD19 negative fraction was stained with CD8 MicroBeads and the positive fraction was collected. Finally, the CD8 negative fraction was stained with CD4 MicroBeads and the positive fraction was retained. Positive fractions were evaluated for purity (S2 Fig) and combined as indicated (Figs 5 and 10), washed two times with PBS and adoptively transferred into naive mice by intravenous injection. The number of each cell type transferred into each mouse was maximized resulting in each mouse from each experiment receiving approximately: CD4+, 1.5x10^6^ cells; CD8+, 3.5x10^6^ cells; CD19+, 5.0x10^6^ cells. In addition, a mix of serum pooled from all 17D-204 immune mice was injected i.p. into naive mice in a volume of 170ul. Twenty-four hours following transfer, mice were challenged with Ang71.

### Statistics

See each table or figure legends for statistical analysis.

## Supporting Information

S1 Fig Serum cytokine levels in vaccinated and non-vaccinated mice infected with wtYFV. (TIF)

S2 Fig Representitive magnetic enrichment profile of lymphocytes/splenocytes from 17D-204 or mock vaccinated mice. Flow cytometry histogram plots of pre-sort or magnet enrichment fractions are displayed. Each fraction was stained for CD19, CD8 and CD4. The percentage displayed represents the total cells in that fraction that are positive for the specific stain as indicated by the placement of the gate.
(TIF)

S3 Fig Expression of CD44 and CD62L on CD11a_hi_ T cells. (TIF)

S4 Fig Analysis of human type I interferon gene expression following 17D vaccination. GEO2 data [8] analysis of type I interferon gene expression in humans following 17D infection (GSE13486-GPL7567) were obtained by the getGEO package in R. A day 0 (D0) group was paired with individual groups for time points; D1, D3, D7 or D21. Each set was combined into a single data frame. The Uniprot.ws package was then used to annotate the gene data specific for interferon alpha and interferon beta genes. Plots are displayed as fold change from day 0 by a 2^logFC transformation. Statistical relevance was determined using the adjusted P value (adj.P.val). No values were significant at a p≤0.1.
(TIFF)
