# Supplementary figures and images for: The 17D-204 Vaccine Strain-Induced Protection against Virulent Yellow Fever Virus Is Mediated by Humoral Immunity and CD4+ but not CD8+ T Cells
Source: PLoS Pathog. 2016 Jul 27;12(7):e1005786. doi: 10.1371/journal.ppat.1005786 (PMC4962991; doi:10.1371/journal.ppat.1005786)

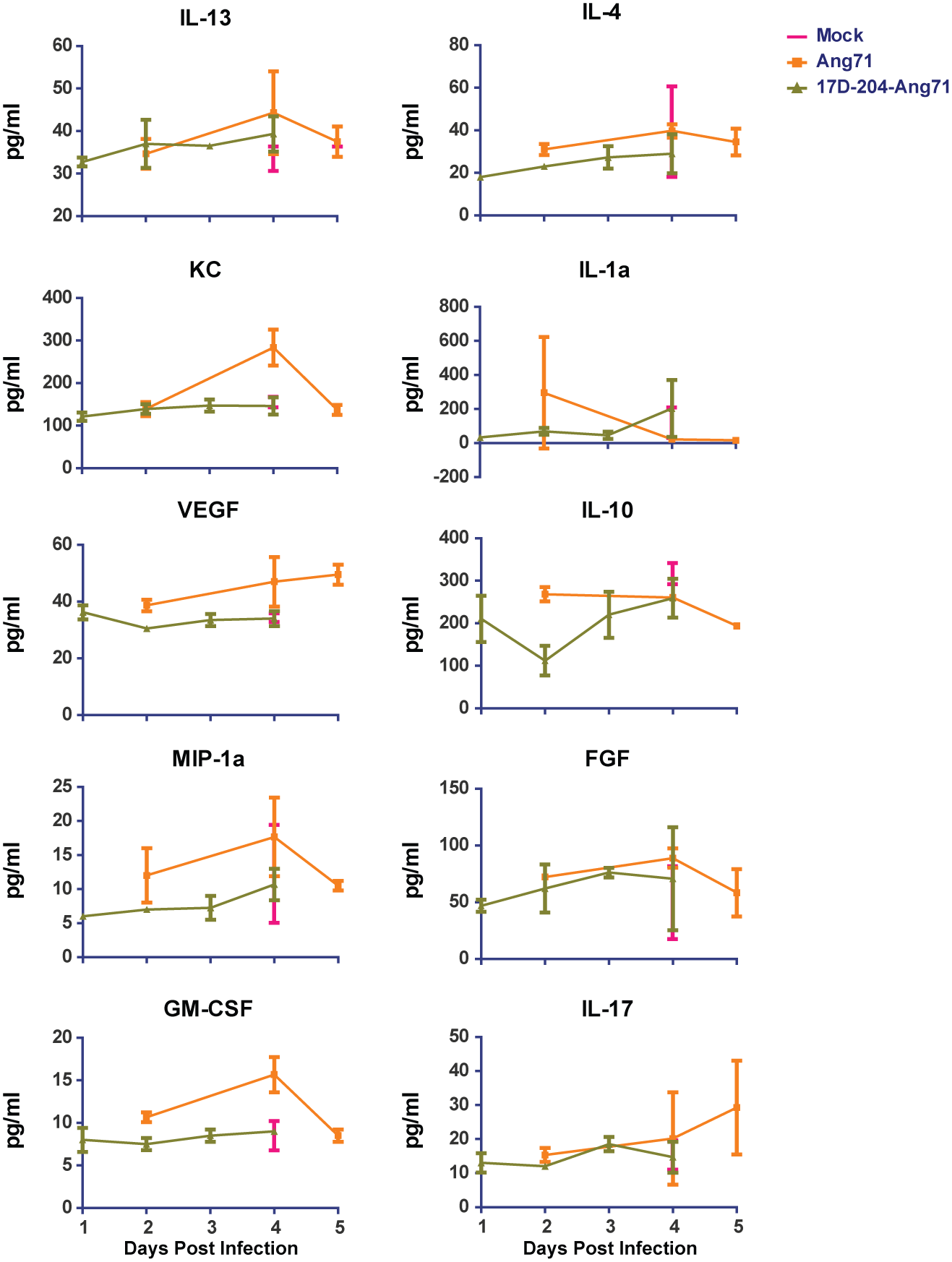

Supplement: S1 Fig — (TIF) [file ppat.1005786.s001.tif]

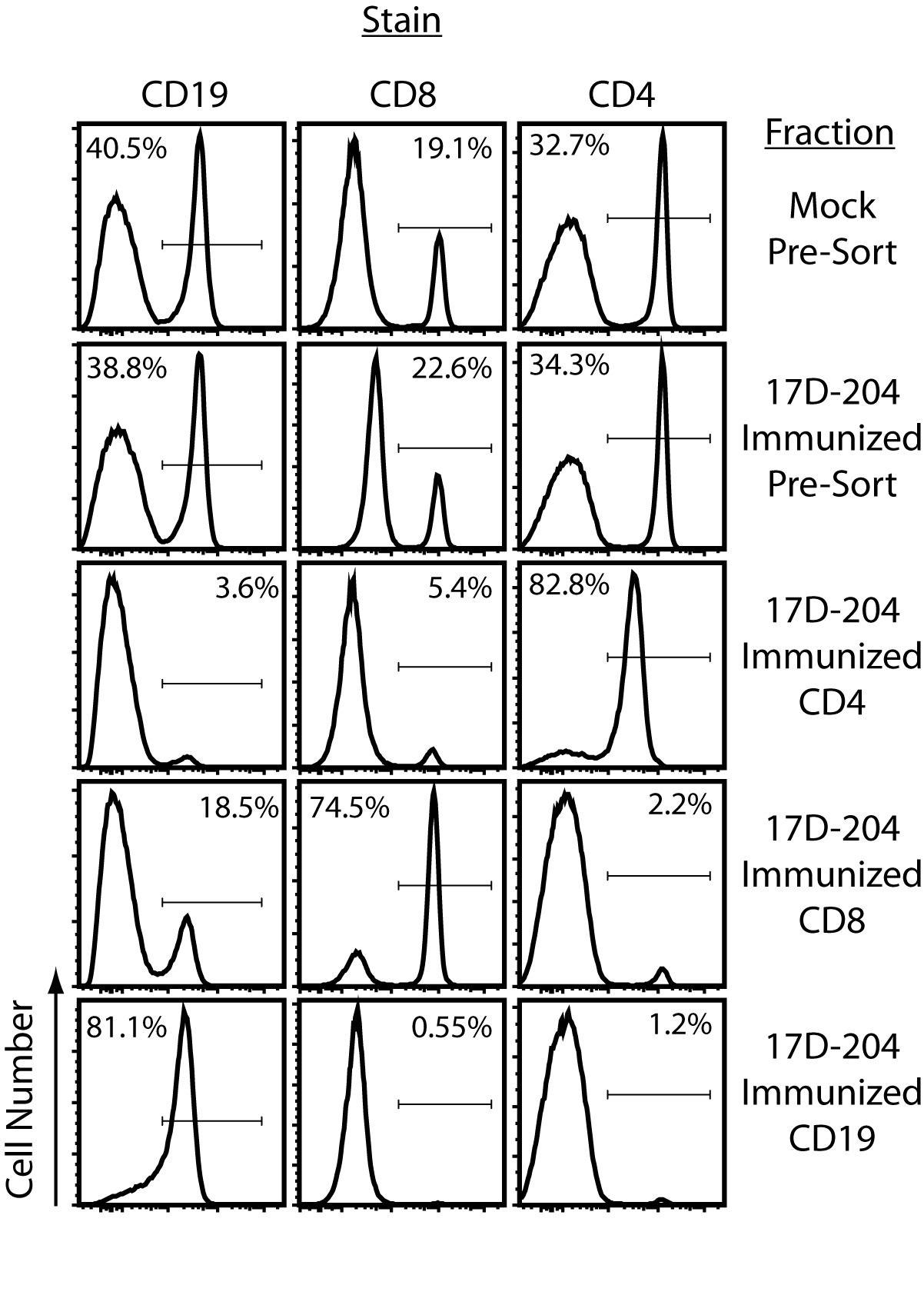

Supplement: S2 Fig — Flow cytometry histogram plots of pre-sort or magnet enrichment fractions are displayed. Each fraction was stained for CD19, CD8 and CD4. The percentage displayed represents the total cells in that fraction that are positive for the specific stain as indicated by the placement of the gate. (TIF) [file ppat.1005786.s002.tif]

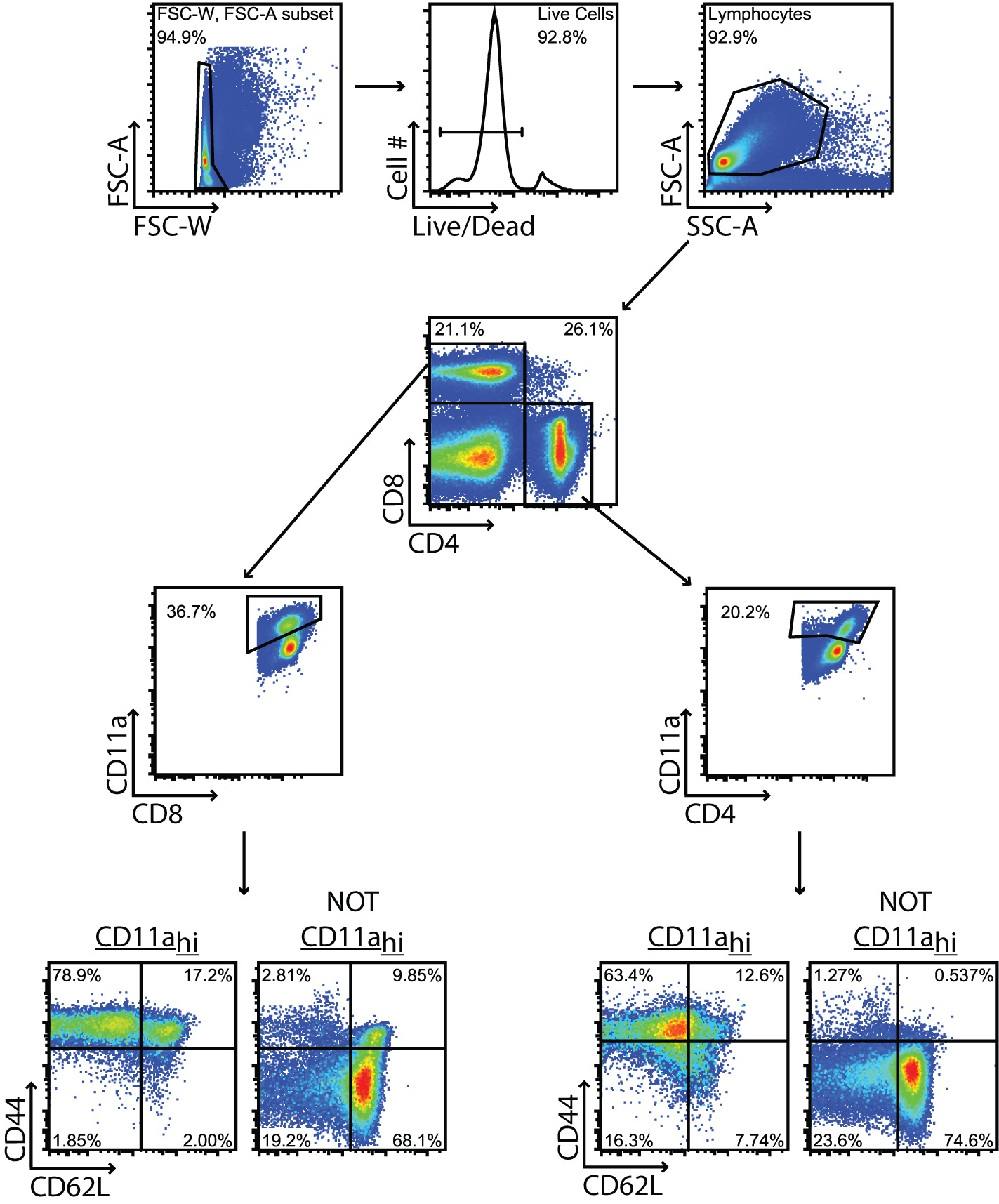

Supplement: S3 Fig — (TIF) [file ppat.1005786.s003.tif]

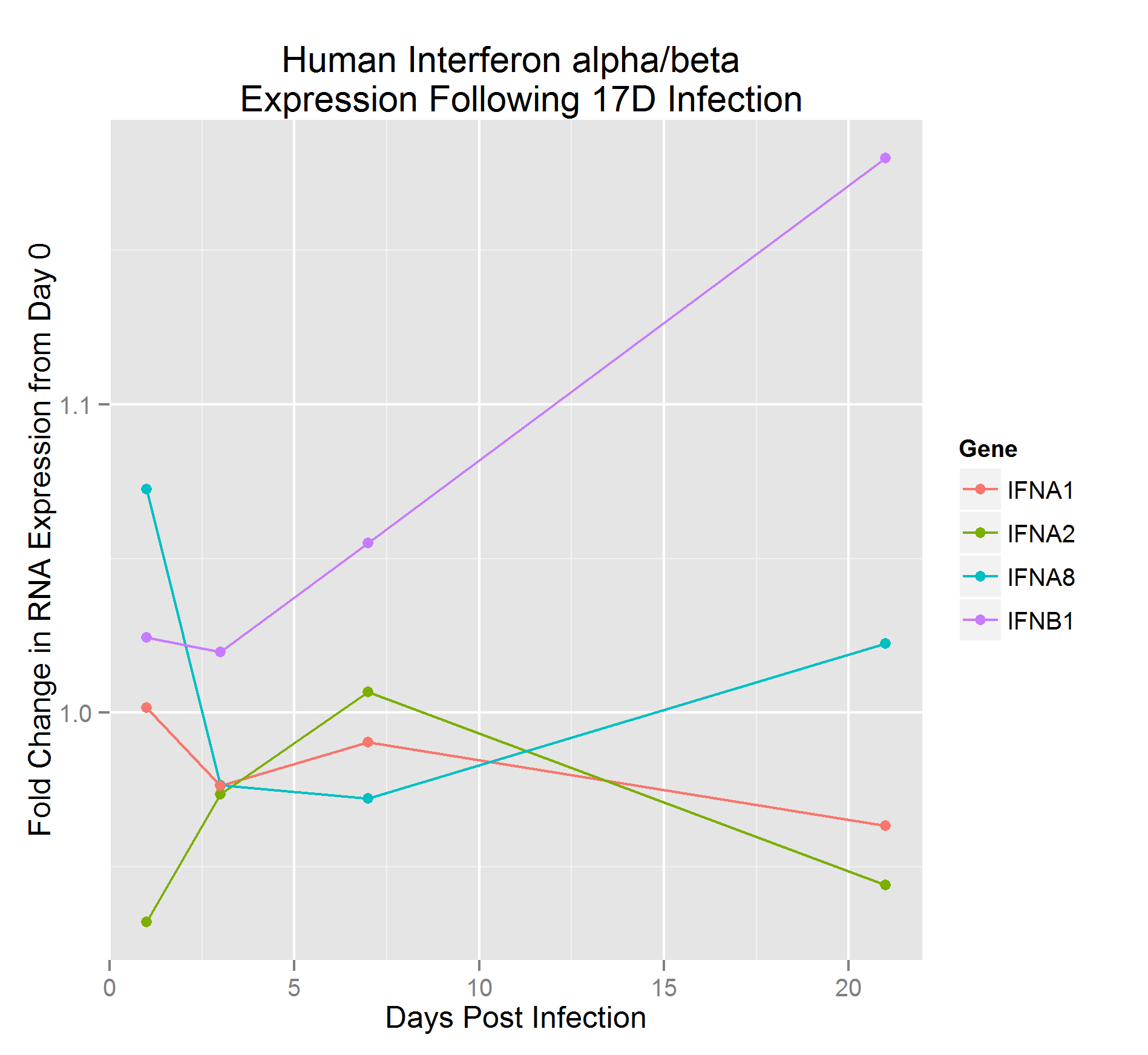

Supplement: S4 Fig — GEO2 data [8] analysis of type I interferon gene expression in humans following 17D infection (GSE13486-GPL7567) were obtained by the getGEO package in R. A day 0 (D0) group was paired with individual groups for time points; D1, D3, D7 or D21. Each set was combined into a single data frame. The Uniprot.ws package was then used to annotate the gene data specific for interferon alpha and interferon beta genes. Plots are displayed as fold change from day 0 by a 2^logFC transformation. Statistical relevance was determined using the adjusted P value (adj.P.val). No values were significant at a p≤0.1. (TIFF) [file ppat.1005786.s004.tiff]
